# Interleukin-2-regulatory T cell axis critically regulates maintenance of hematopoietic stem cells

**DOI:** 10.18632/oncotarget.16377

**Published:** 2017-03-18

**Authors:** Sabrina Giampaolo, Gabriela Wójcik, Edgar Serfling, Amiya K. Patra

**Affiliations:** ^1^ Department of Molecular Pathology, Institute of Pathology, University of Wrzburg, Wrzburg, Germany; ^2^ Institute of Translational and Stratified Medicine, Peninsula Schools of Medicine and Dentistry, University of Plymouth, Plymouth, UK; ^3^ Comprehensive Cancer Center Mainfranken, University of Wrzburg, Wrzburg, Germany

**Keywords:** hematopoietic stem cells, IL-2, Treg cells, IL-10, IFN-γ, Immunology and Microbiology Section, Immune response, Immunity

## Abstract

The role of IL-2 in HSC maintenance is unknown. Here we show that *Il2*^−/−^ mice develop severe anomalies in HSC maintenance leading to defective hematopoiesis. Whereas, lack of IL-2 signaling was detrimental for lympho- and erythropoiesis, myelopoiesis was enhanced in *Il2*^−/−^ mice. Investigation of the underlying mechanisms of dysregulated hematopoiesis in *Il2*^−/−^ mice shows that the IL-2-T_reg_ cell axis is indispensable for HSC maintenance and normal hematopoiesis. Lack of T_reg_ activity resulted in increased IFN-? production by activated T cells and an expansion of the HSCs in the bone marrow (BM). Though, restoring T_reg_ population successfully rescued HSC maintenance in *Il2*^−/−^ mice, preventing IFN-? activity could do the same even in the absence of T_reg_ cells. Our study suggests that equilibrium in IL-2 and IFN-? activity is critical for steady state hematopoiesis, and in clinical conditions of BM failure, IL-2 or anti-IFN-? treatment might help to restore hematopoiesis.

## INTRODUCTION

In vertebrates, postnatal hematopoiesis mainly occurs in the bone marrow (BM). Through a complex but precisely regulated differentiation process the hematopoietic stem cells (HSC) give rise to mature blood cells of all lineages. HSC niche-derived signals, originating from cells as diverse as the osteoblasts, endothelial cells, Cxcl12-abundant reticular (CAR) cells, Nestin^+^ mesenchymal stem cells etc., and a host of cell-intrinsic factors, such as Notch1, Runx1, GATA2, and SCL etc. play a key role in the maintenance of HSCs in the BM [1–10]. HSCs possess the unique traits of quiescence, and the ability to self-renew, which are absolutely critical for the general well-being and longevity of any organism as HSCs are the sole source for replenishment of all types of blood cells in case of demands that arise under various physiological and pathological conditions [11, 12].

Phenotypically HSCs are characterized as the lineage marker-negative (Lin-), stem cell antigen-1 (Sca1) and the stem cell factor receptor (SCFR or c-Kit) positive (Lin-Sca1^+^c-Kit^+^: LSK) cells in the BM. Various *in vivo* and *in vitro* analyses of LSK cells identified two distinct populations, the long- and the short-term repopulating stem cells (LT-HSC and ST-HSC). The LT-HSCs, which do not express the fetal liver kinase-2 (Flk2) on their surface, retain the ability for life-long self-renew and at the same time give rise to the ST-HSCs, whereas the Flk2 expressing ST-HSCs are capable of limited self-renewal and give rise to the multipotent progenitor cells for all blood lineages [13]. Maintenance of HSC quiescence is vital to prevent exhaustion and thereby for the integrity of the hematopoietic system [14, 15]. Cytokines and growth factors form an important class of regulatory molecules, which are indispensable for the maintenance of HSC quiescence [16–21]. Though, recent studies have identified many such cytokines, chemokines and other hematopoietic growth factors, a role for the primarily lymphocyte-specific growth factor interleukin-2 (IL-2) has not been identified.

In T cells, IL-2 binds to a heterotrimeric receptor consisting of the IL-2 receptor α (IL-2Rα, CD25), IL-2Rβ (CD122) and the common γ (CD132, γ_c_) chain [22, 23]. Upon ligation, IL-2 activates the receptor bound tyrosine kinases Jak1 and Jak3, which in turn phosphorylate the signal transducer and activator of transcription 5 (STAT5) transcription factors (TF) [24–26]. Activated STAT5 subsequently regulate the expression of various target genes that executes IL-2 signals [27–29]. IL-2 is mainly produced by activated CD4^+^ and CD8^+^ T cells, and plays a critical role in the survival and proliferation of lymphocytes [22, 23, 30–33]. Recent studies have established the indispensable role of IL-2 in the maintenance of the CD4^+^CD25^+^Foxp3^+^ regulatory T (T_reg_) cells, which are vital to keep immune homeostasis in place [23, 34, 35]. Lack of IL-2 signaling results in the death of T_reg_ cells leading to rapid development of various autoimmune disorders, such as lymphoproliferation, colitis etc., [36–39]. A similar phenotype has also been reported for mice deficient in Foxp3 or any component of IL-2 signaling pathway [30, 40]. In addition, humans having an inactivating mutation in the Foxp3 gene suffer from a severe autoimmune pathology called IPEX (Immunodysregulation Polyendocrinopathy Enteropathy X-linked) syndrome [41]. Thus, the role of IL-2 in T cell function and in maintaining immune homeostasis is well established.

We have recently reported that IL-2 plays a critical role in maintaining erythropoiesis by modulating T_reg_ cell activity in the BM [42]. However, the influence of IL-2 on HSC generation or maintenance in the BM has not yet been investigated. In this study we have analysed whether lack of IL-2 signaling has any influence on hematopoiesis and report that the IL-2-T_reg_-T_eff_ cell axis plays an indispensable role in maintaining steady state HSC population in the BM. Deficiency in IL-2 signaling results in an IFN-γ-mediated disruption of equilibrium in HSC physiology leading to impaired hematopoiesis, which might be a major contributing factor towards the severe phenotype observed in all IL-2 signaling deficient mice, and most likely also in humans.

## RESULTS

### Impaired HSC maintenance in *Il2*^−/−^ mice

**Figure 1:**
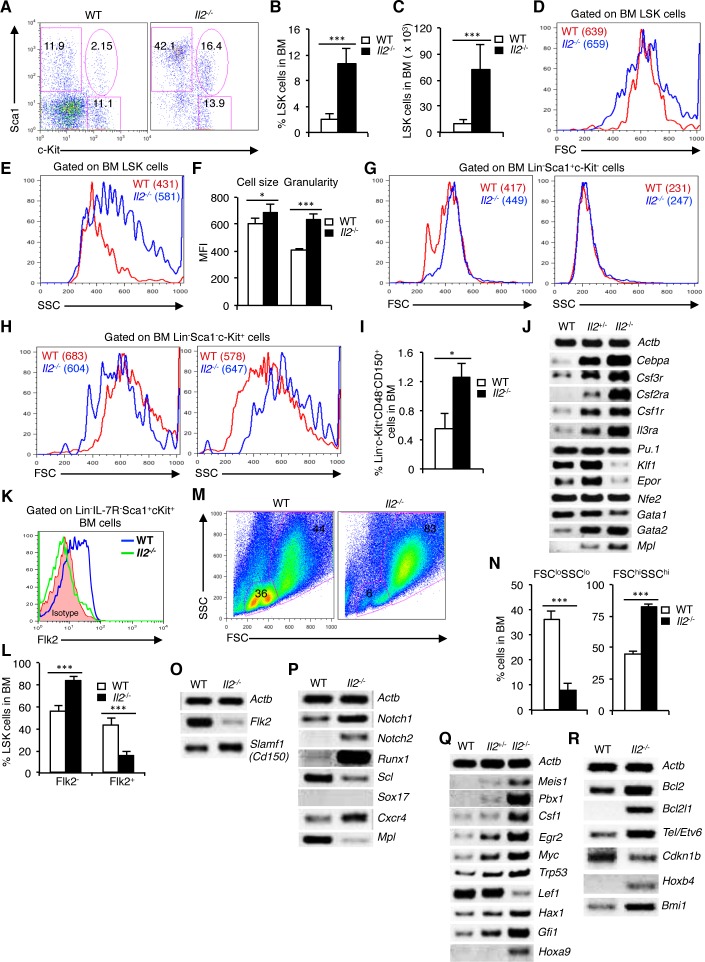
Impaired maintenance of HSCs in *Il2*^−/−^ mice BM **A**. Flow cytometry analysis of BM cells for LSK population in WT and *Il2*^−/−^ mice. **B**. Proportion of LSK cells in the BM of WT and *Il2*^−/−^ mice (*n* = 20 per group). **C**. LSK cells distribution in absolute numbers in WT and *Il2*^−/−^ mice (*n* = 20 per group). **D**. Cell size of WT and *Il2*^−/−^ HSCs as reflected by their FSC distribution pattern. **E**. SSC distribution pattern of WT and *Il2*^−/−^ HSCs. **F**. Mean cell size and granularity of WT and *Il2*^−/−^ HSCs. **G**. FSC and SSC profiles of BM Lin^−^Sca1^+^c-Kit^−^ cells from WT and *Il2*^−/−^ mice. **H**. Profiles of WT and *Il2*^−/−^ BM Lin^−^Sca1^−^c-Kit^+^ cells according to their FSC and SSC distribution pattern. **I**. Proportion of Lin^−^c-Kit^+^CD48^−^CD150^+^ cell population in the BM from WT and *Il2*^−/−^ mice (*n* = 5 per group). **J**. Myeloid, lymphoid and erythroid lineage-specific genes expression in WT, *Il2*^+/−^ and *Il2*^−/−^ mice. **K**. Flow cytometry revealing the distribution of LT-HSCs and ST-HSCs among LSK cells in indicated mice (*n* = 5 for WT and 4 for *Il2*^−/−^ mice). **L**. Population distribution of LT- and ST-HSC in the BM of WT and *Il2*^−/−^ mice based on Flk2 expression (*n* = 7 per group). **M**. Flow cytometry profiles of BM cells in WT and *Il2*^−/−^ mice according to their FSC and SSC distribution pattern (*n* = 20 per group). **N**. Quantification of FSC^lo^SSC^lo^ and FSC^hi^SSC^hi^ cells in WT and *Il2*^−/−^ mice. **O**. RT-PCR analysis of LT- and ST-HSC-specific genes expression in sorted LSK cells from WT and *Il2*^−/−^ mice. **P**. Semi-quantitative RT-PCR on WT and *Il2*^−/−^ LSK cells for critical genes involved in HSC maintenance. **Q**. Gene expression analysis of TFs and signaling molecules in LSK cells from WT, *Il2*^+/−^ and *Il2*^−/−^ mice. **R**. Cell survival and HSC proliferation-specific genes expression in LSK cells from WT, *Il2*^+/−^ and *Il2*^−/−^ mice. Numbers inside each dot plot represent percent respective population, and in histograms represent the mean fluorescence intensity (MFI). Data are representative of 2-5 independent experiments and shown as mean ± s.d., in (B, C & N) ***P < 0.0001, in (F) *P = 0.0389, ***P = 0.0001, in (I) *P = 0.0348, and in (L) ***P = 0.0002, unpaired *t*-test.

To investigate whether IL-2 deficiency affects hematopoiesis, we first of all analysed the distribution of HSCs in *Il2*^−/−^ mice. Evaluation of BM cells revealed a strong increase in LSK population in *Il2*^−/−^ mice compared to WT mice (Figure 1A). The increased LSK population in *Il2*^−/−^ mice was gender-independent and was observed both in proportion (Figure 1B) and in absolute numbers (Figure 1C). Interestingly, though the *Il2*^−/−^ LSK cells were slightly bigger in size (Figure 1D, 1F), they showed a markedly higher granularity compared to WT LSK cells (Figure 1E, 1F). We also observed a consistently strong increase in Lin-Sca1^+^c-Kit^−^ (Sca1^+^) cells in *Il2*^−/−^ mice, whereas the distribution of Lin^−^Sca1^−^c-Kit^+^ (c-Kit^+^) cells was similar compared to WT mice (Figure 1A). However, the increase in LSK cells in *Il2*^−/−^ mice was independent of the impairment in Sca1^+^ population in these mice. Compared to LSK cells, *Il2*^−/−^ Sca1^+^ and c-Kit^+^ cells showed distinct patterns in their cell size and granularity, comparable with that of WT mice (Figure 1G, 1H and Supplementary Figure 1A, B). In addition, HSC analysis in BM using CD48 and CD150 as markers also showed an increase in HSC (Lin^−^c-Kit^+^CD48^−^CD150^+^) population in the *Il2*^−/−^ mice compared to WT mice (Figure 1I). This further ruled out any possible contamination from cells expressing Sca1 in the perturbed HSC (LSK) population in *Il2*^−/−^ mice. Similar to BM, impaired HSC distribution was also observed in the spleen of *Il2*^−/−^ mice suggesting a systemic defect in HSC maintenance in the absence of IL-2 signaling (Supplementary Figure 1C, D).

Analysis of mature hematopoietic cell populations in WT and *Il2*^−/−^ mice revealed a drastically altered distribution of lymphoid (CD3^+^, B220^+^ and NK1.1^+^), myeloid (CD11b^+^, CD11c^+^, Gr1^+^ and F4/80^+^) and erythroid (Ter119^+^ and CD41^+^) lineage cells in *Il2*^−/−^ mice BM (Supplementary Figure 1E). In general, in *Il2*^−/−^ mice myelopoiesis was strongly increased over the impaired lympho- and erythropoiesis, indicating a myeloid bias of the *Il2*^−/−^ HSC compared to WT controls (Supplementary Figure 1E). This myeloid-biased hematopoiesis was supported by an increased expression of myeloid-specific genes (*Cebpa, Csf3r, Csf2ra, Csf1r* and *Il3ra*) in the *Il2*^−/−^ mice (Figure 1J). To dissect this severely perturbed hematopoiesis, we next investigated whether the distribution of the LT- and the ST-HSCs are altered in the context of IL-2 deficiency. Intriguingly, analysis of Flk2 expression revealed a significant increase in Flk2^−^ LT-HSC and a corresponding decrease in the Flk2^+^ ST-HSC in *Il2*^−/−^ mice compared to that in WT mice (Figure 1K, 1L). Moreover, the defective hematopoiesis in absence of IL-2 signaling was readily evident from the forward (FSC) and side scatter (SSC) distribution of the BM cells as a clear loss of FSC^lo^SSC^lo^ and an increase in FSC^hi^SSC^hi^ cells was observed in *Il2*^−/−^ mice compared to the WT mice (Figure 1M, 1N). The impairment in *Il2*^−/−^ HSC was further confirmed as we observed a suppressed *Flk2* and an enhanced *Slamf1* (*Cd150*) expression in the LSK cells from *Il2*^−/−^ mice, which was exactly opposite in the WT LSK cells (Figure 1O). Previous studies have implicated the criticality of Notch signaling and the Runx TFs in the generation and proliferation of HSC [7]. Interestingly, a strong increase in *Notch* (*Notch1* and *Notch2*) and *Runx1* expression was evident in the *Il2*^−/−^ LSK cells suggesting that enhanced Notch-Runx1 activity could be the factor promoting dysregulated HSC maintenance in *Il2*^−/−^ mice (Figure 1P). Additionally, in *Il2*^−/−^ mice the expression of *Scl, Cxcr4* and *cMpl*, the key signalling molecules regulating HSC maintenance were also altered [9, 10, 18, 43–46] (Figure 1P). Further, analysis of TFs involved in HSC quiescence revealed a dose-dependent increase in their expression in *Il2*^−/−^ HSCs [47–56] compared to WT cells (Figure 1Q). Interestingly, the *Il2*^−/−^ LSK cells expressed higher levels of *Bcl2* and *Bcl2l1*, indicating that the dysregulated hematopoiesis in *Il2*^−/−^ mice was not due to the death of HSCs (Figure 1R). Also, in line with the increased number of LSK cells, expression of the cell cycle inhibitor p27^kip1^ (*Cdkn1b*) was strongly downregulated and genes regulating HSC expansion were enhanced in the *Il2*^−/−^ mice (Figure 1R).

Next, we investigated whether the HSC defects observed in *Il2*^−/−^ mice are replicated in all mice that lack IL-2 signaling. Analysis of *Il2ra*^−/−^ or *Jak3*^−/−^ mice showed a similar increase in LSK cells, and a loss of FSC^lo^SSC^lo^ BM cells with corresponding increase in FSC^hi^SSC^hi^ cells as was observed in *Il2*^−/−^ mice suggesting a specific influence of lack of IL-2 signaling on HSC maintenance (Supplementary Figure 1F-K).

### IL-2 signaling influences hematopoiesis

**Figure 2:**
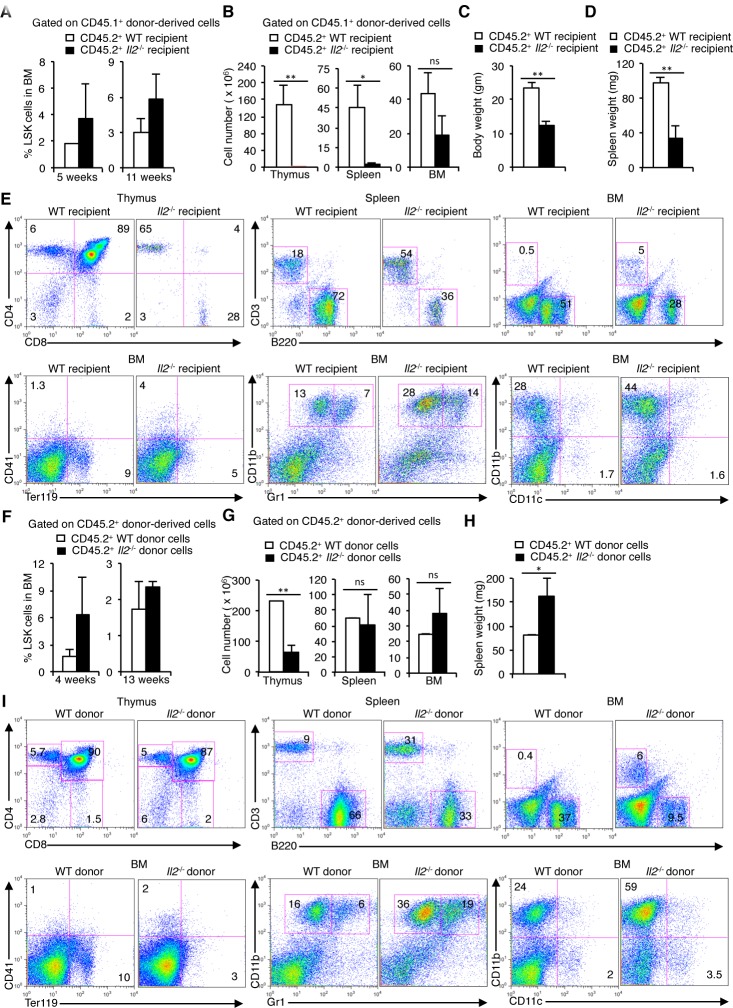
HSC maintenance in BM depends on IL-2 signaling **A**. Enumeration of CD45.1^+^ donor-derived LSK cells distribution 5 and 11 weeks post-transfer in the BM of irradiated CD45.2^+^ WT or *Il2*^−/−^ recipient mice. **B**. Number of CD45.1^+^ donor-derived cells in the thymus, spleen and BM of irradiated CD45.2^+^ WT or *Il2*^−/−^ recipient mice 5 weeks after transfer. **C**. Evaluation of body weight, and **D**. spleen weight of the recipient mice 5 weeks after cell transfer. **E**. Flow cytometry profiles of the distribution of various donor-derived mature hematopoietic cell populations in the thymus, spleen and BM of recipient mice. Data are representative of two independent experiments (*n* = 4 per group/experiment). **F**. Distribution of CD45.2^+^ WT or *Il2*^−/−^ donor-derived LSK cells distribution 4 and 13 weeks post-transfer in the BM of irradiated CD45.1^+^ WT recipient mice. **G**. Number of CD45.2^+^ WT or *Il2*^−/−^ donor-derived cells in the Thymus, spleen and BM of irradiated CD45.1^+^ WT recipient mice 4 weeks after transfer. **H**. Spleen weight of the recipient mice 4 weeks after cell transfer. **I**. Flow cytometry profiles of the distribution of various CD45.2^+^ WT or *Il2*^−/−^ donor-derived mature hematopoietic cell populations in the thymus, spleen and BM of CD45.1^+^ WT recipient mice. Data are representative of two independent experiments (*n* = 4 per group/experiment). Numbers inside each FACS plot represent percent respective population. Data are shown as mean ± s.d., in (B) **P = 0.0090, *P = 0.0176, (C) **P = 0.0034, (D) **P = 0.0096, (G) **P = 0.0028, (H) *P = 0.0198, and ns = not significant, unpaired *t*-test.

To explore if IL-2 signals influence HSC maintenance, we adoptively transferred CD45.1^+^ WT BM cells to lethally irradiated CD45.2^+^ WT or *Il2*^−/−^ mice and analysed donor-derived cells-specific hematopoiesis in the recipient mice. Analysis of LSK cells in the BM 5 weeks post-transfer revealed an increase in donor-derived LSK cells in the *Il2*^−/−^ recipients compared to the WT recipients replicating a phenotype observed in *Il2*^−/−^ mice (Figure 2A). This pattern persisted even at 11 weeks after BM cell transfer (Figure 2A), suggesting a disturbed HSC maintenance in an IL-2-deficient environment. In addition, similar to *Il2*^−/−^ mice, *Il2*^−/−^ recipients showed increased Sca1^+^ cells in the BM both at 5 and 11 weeks after cell transfer (Supplementary Figure 2A). Further, the thymus, spleen and BM cellularity in the *Il2*^−/−^ recipients was severely reduced compared to WT recipients indicating an inefficient hematopoietic reconstitution in the *Il2*^−/−^ recipients despite having higher number of HSCs (Figure 2B). At 5 weeks, the *Il2*^−/−^ recipients were consistently underweight (Figure 2C), and their spleens were smaller in size (Figure 2D) compared to WT recipients. Analysis of lineage-positive cells in lymphoid organs demonstrated a marked defect in lymphopoiesis and erythropoiesis, and replicated the myeloid bias of HSCs in an IL-2-deficient environment similar to that observed in the *Il2*^−/−^ mice (Figure 2E). Though, the thymic, splenic and BM cellularity improved at 11 weeks after transfer, the *Il2*^−/−^ recipients still remained underweight (Supplementary Figure 2B, C). However, the increase in cellularity was not due to a normal hematopoiesis rather it was the outcome of the dysregulated hematopoiesis as shown in Figure 2E, with myeloid cells being the major population in these organs. Also, due to impaired hematopoiesis splenomegaly was observed at 11 weeks after cell transfer in all *Il2*^−/−^ recipients (Supplementary Figure 2D).

To further clarify the role of IL-2 in HSC maintenance and lineage differentiation, we transferred CD45.2^+^ WT or *Il2*^−/−^ mice BM cells to lethally irradiated CD45.1^+^ WT hosts. Analysis at 4 weeks post-transfer again revealed an increased LSK cell population in the *Il2*^−/−^ BM transferred WT recipients compared to the WT BM transferred mice (Figure 2F). At this time point an increased Sca1^+^ population, characteristic of the *Il2*^−/−^ mice was also observed in the *Il2*^−/−^ BM transferred WT recipients (Supplementary Figure 2E). Analysis of cellularity in the lymphoid organs revealed that *Il2*^−/−^ BM transferred WT recipients had strongly reduced thymocyte numbers, whereas spleen and BM cell numbers were comparable with that in WT BM transferred WT recipients (Figure 2G). However, again, the comparable cellularity in spleen and BM was due to impaired hematopoiesis, as all *Il2*^−/−^ BM transferred WT recipients showed significantly enlarged spleen (Figure 2H), and in the BM, myelopoiesis was enhanced over lympho- and erythropoiesis (Figure 2I). This was significant as in an IL-2-sufficient environment even with increased number of LSK cells, *Il2*^−/−^ BM cells failed to efficiently reconstitute the hematopoietic system.

Interestingly, 13 weeks after BM transfer the HSC population in the *Il2*^−/−^ BM transferred WT recipients were similar to that in the WT BM transferred WT recipients (Figure 2F), which in turn gave rise to efficient hematopoietic reconstitution resulting in comparable cellularity, normal body weight as well as spleen size (Supplementary Figure 2F-H). Also, the myeloid-biased hematopoiesis in IL-2-deficient background was largely reversed (Supplementary Figure 2I). The contrasting observations at an early (4 weeks) compared to a late time point (13 weeks) after BM cell transfer suggests that the reconstitution capacity of the *Il2*^−/−^ HSCs is significantly impaired relative to the WT HSCs and require prolonged sensitization in an IL-2-sufficient environment in order to promote normal hematopoiesis. Altogether, both sets of experiments underline the critical requirement of IL-2 signaling for HSC maintenance and normal hematopoiesis.

### Hematopoietic defects in *Il2*^−/−^ mice are T cell-mediated

**Figure 3:**
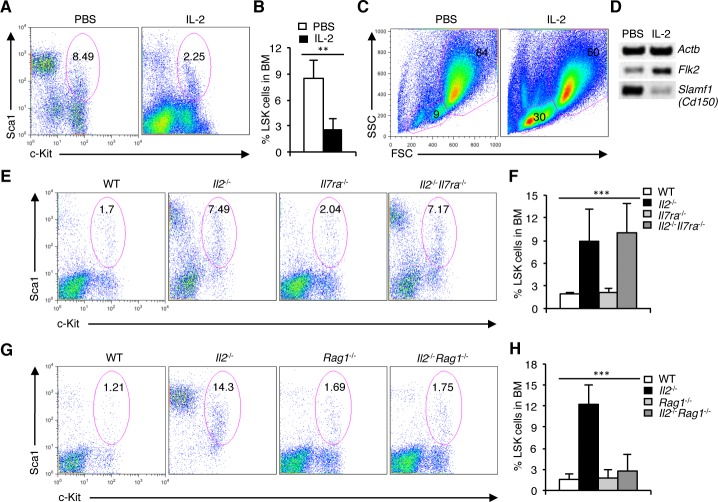
Defective HSC maintenance in *Il2*^−/−^ mice is T cell-mediated **A**. Distribution of LSK cells in the BM of PBS or IL-2 treated *Il2*^−/−^ mice. **B**. Proportion of LSK cells in the BM of *Il2*^−/−^ mice treated either with PBS or IL-2. **C**. FSC/SSC distribution of BM cells from PBS or IL-2 treated *Il2*^−/−^ mice. **D**. RT-PCR analysis of *Flk2* and *Slamf1* expression in sorted BM LSK cells from *Il2*^−/−^ mice treated either with PBS or IL-2. **E**. Flow cytometry analysis of LSK cells distribution in the BM of WT, *Il2*^−/−^, *Il7ra*^−/−^ and *Il2*^−/−^*Il7ra*^−/−^ mice. **F**. Quantification of the distribution of BM LSK population in indicated mice. **G**. Distribution of LSK cells in the BM of WT, *Il2*^−/−^, *Rag1*^−/−^ and *Il2*^−/−^*Rag1*^−/−^ mice. **H**. Proportion of LSK cells in BM cells from WT, *Il2*^−/−^, *Rag1*^−/−^ and *Il2*^−/−^*Rag1*^−/−^ mice. Numbers inside each FACS plot represent percent respective population. Data are representative of 3 independent experiments, (*n* = 4 per group) and shown as mean ± s.d., in (F) ***P = 0.0004 and (H) ***P < 0.0001, one-way ANOVA.

To show that IL-2 signaling directly influences HSC maintenance, we treated *Il2*^−/−^ mice with exogenous IL-2. Surprisingly, IL-2 treatment significantly reversed the altered pattern in HSC distribution in *Il2*^−/−^ mice, which was now comparable to that in WT mice (Figure 3A, 3B). IL-2 treatment restored the missing FSC^lo^SSC^lo^ population in the BM (Figure 3C), and eventually led to resolving the defective hematopoiesis in *Il2*^−/−^ mice (Supplementary Figure 3A). Also, IL-2 treatment reversed the distribution of HSC subpopulations, as it enhanced *Flk2* and reduced *Slamf1* expression in *Il2*^−/−^ LSK cells (Figure 3D). These data show that IL-2 signals exert a beneficial influence on HSC maintenance that is essential for normal hematopoiesis.

Next, to investigate the basis of this strong effect of IL-2 signaling on HSC maintenance, we hypothesized that the massive T-lymphocyte proliferation reported in *Il2*^−/−^ mice could in some way be responsible for the hematopoietic defects in these mice [30, 57]. To verify this issue, we analysed *Il2*^−/−^*Il7ra*^−/−^ mice in which lymphoproliferation was effectively prevented [42]. However, analysis of LSK cells in these mice showed, the persistence of HSC defects both in the BM and in spleen as was in the *Il2*^−/−^ mice (Figure 3E, 3F and Supplementary Figure 3B, C). To resolve this intriguing observation we hypothesized that most likely the residual T-lymphocytes do not function normally in the absence of IL-2 signaling and thereby induced the HSC defects in the *Il2*^−/−^*Il7ra*^−/−^ mice. To investigate this possibility, we bred *Il2*^−/−^ mice with *Rag1*^−/−^ mice in order to abolish all T cells. Surprisingly, the *Il2*^−/−^*Rag1*^−/−^ mice demonstrated a normal LSK population both in the BM (Figure 3G, 3H) and in the spleen (Supplementary Figure 3D, E), as well as a normal hematopoiesis in contrast to the *Il2*^−/−^ mice. These observations suggest that the defective HSC maintenance and dysregulated hematopoiesis in the *Il2*^−/−^ mice are to a large part mediated by T cells.

### T_reg_ cell activity is indispensable for HSC integrity

**Figure 4:**
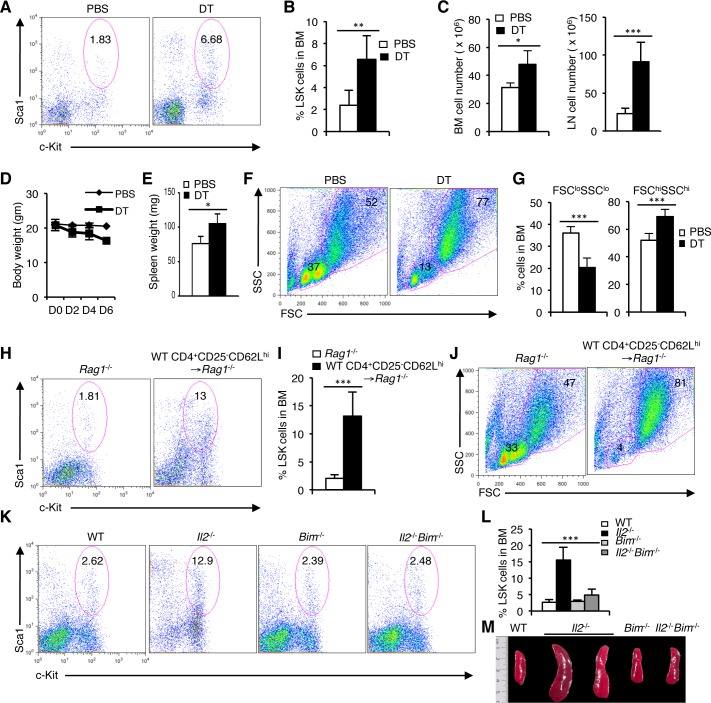
T_reg_ cell activity is critical for steady state hematopoiesis **A**. LSK cells distribution in the BM of DEREG mice treated with PBS or diphtheria toxin (DT). **B**. Quantification of the LSK population distribution in DEREG mice BM after PBS or DT treatment. **C**. Cellularity in the BM and LNs of DEREG mice following PBS or DT treatment. **D**. Evaluation of body weight following each PBS or DT injection to the DEREG mice. **E**. Spleen weight of DEREG mice treated with PBS or DT. **F**. FSC/SSC distribution of BM cells from PBS or DT treated DEREG mice. **G**. Evaluation of FSC^lo^SSC^lo^ and FSC^hi^SSC^hi^ BM cells in PBS or DT treated DEREG mice. **H**. Distribution of LSK population in the BM of *Rag1*^−/−^ mice, 3 weeks after transfer of WT CD4^+^CD25^−^CD62L^hi^ cells, compared to normal WT or *Rag1*^−/−^ mice. **I**. Quantification of BM LSK cells in *Rag1*^−/−^ mice, 3 weeks post-transfer of WT CD4^+^CD25^−^CD62L^hi^ cells in comparison to control *Rag1*^−/−^ mice. **J**. FSC/SSC distribution of BM cells from WT CD4^+^CD25^−^CD62L^hi^ cells transferred *Rag1*^−/−^ mice compared to control *Rag1*^−/−^ mice. **K**. Flow cytometry profiles of LSK cells in BM of WT, *Il2*^−/−^, *Bim*^−/−^ and *Il2*^−/−^*Bim*^−/−^ mice. **L**. Quantification of the distribution of BM LSK population in indicated mice. **M**. Photograph of spleens from WT, *Il2*^−/−^, *Bim*^−/−^ and *Il2*^−/−^*Bim*^−/−^ mice. Numbers inside each FACS plot represent percent respective population. Data are shown as mean ± s.d., in (B) **P = 0.0043, (C) *P = 0.0113 and ***P = 0.0005, (E) *P = 0.01734, (G) ***P = 0.0003, ***P = 0.0008, (I) ***P = 0.0006, and in (L) ***P < 0.0001 unpaired *t*-test. Data are representative of 3 independent experiments, (*n* = 4 per group).

The persistence of HSC defects in the *Il2*^−/−^*Il7ra*^−/−^ but not in *Il2*^−/−^*Rag1*^−/−^ mice raised the question of how T cell activity could influence hematopoiesis in the BM. IL-2 signaling has been reported to be indispensable for T_reg_ cell survival and accordingly, *Il2*^−/−^ mice show a strongly reduced T_reg_ cell population (Supplementary Figure 4A). We have shown previously that in the *Il2*^−/−^*Il7ra*^−/−^ mice T_reg_ cell population is also drastically reduced [42]. To prove that the lack of adequate number of T_reg_ cells in the *Il2*^−/−^*Il7ra*^−/−^ and *Il2*^−/−^ mice influences HSC maintenance and hematopoiesis, we adopted an inducible T_reg_ cell depletion model [58]. Treatment of DEREG mice with diphtheria toxin (DT) rapidly depleted T_reg_ cells in these mice creating a situation similar to the *Il2*^−/−^ mice (Supplementary Figure 4B). Analysis of LSK cells in the DT-treated DEREG mice revealed a strong increase compared to the control PBS-treated mice (Figure 4A, 4B). Due to defective hematopoiesis in DT-treated DEREG mice cellularity in BM and peripheral lymphoid organs were enhanced compared to the control mice (Figure 4C). Similar to the *Il2*^−/−^ mice, DT-treated mice, consistently lost body weight (Figure 4D), and showed enlarged spleen (Figure 4E) very quickly after DT treatment indicating overall defects in hematopoiesis following depletion of T_reg_ cells. These defects were readily evident in the BM as a strong reduction in FSC^lo^SSC^lo^ cells and an increase in FSC^hi^SSC^hi^ cells was observed in DT-treated animals compared to the control animals (Figure 4F, 4G). Ablation of T_reg_ cell activity also enhanced the LSK population in the spleen further resembling the hematopoietic defects observed in the *Il2*^−/−^ mice (Supplementary Figure 4C, D). Analysis of lineage-positive cells in the BM further supported the critical role of T_reg_ cells in hematopoiesis as lack of T_reg_ cell activity in the DT-treated DEREG mice promoted myelopoiesis (Supplementary Figure 4E-H), an outcome similar to that in the *Il2*^−/−^ mice. These observations suggest that in the absence of T_reg_ cells most likely the activated effector T cells (T_eff_) activity was responsible for the hematopoietic defects observed in the IL-2 signaling-deficient mice.

To further consolidate our observation regarding the essentiality of T_reg_ activity in HSC maintenance, we adoptively transferred T_reg_-depleted CD4^+^ T_eff_ cells to *Rag1*^−/−^ mice. In contrast to control mice, T_eff_ cells transferred *Rag1*^−/−^ mice rapidly developed a hematopoietic phenotype similar to that of the *Il2*^−/−^ mice. A strong increase in the BM LSK population (Figure 4H, 4I), concomitant with a decrease in FSC^lo^SSC^lo^ BM cells (Figure 4J) reflected the severity of defects in HSC maintenance and hematopoiesis when T_reg_ cell activity was lacking.

Next we investigated whether restoration of T_reg_ cell activity can reverse the defects in HSC maintenance and facilitate normal hematopoiesis in the *Il2*^−/−^ mice. Analysis of *Il2*^−/−^*Bim*^−/−^ mice in which Bim deficiency completely restored T_reg_ population [42], showed normal hematopoiesis even if IL-2 signaling was still absent. In contrast to the *Il2*^−/−^ mice, LSK population in BM (Figure 4K, 4L), and spleen (Supplementary Figure 5A, B) of the *Il2*^−/−^*Bim*^−/−^ mice was comparable to that of the control mice. As an immediate sign of normal hematopoiesis, the FSC/SSC distribution of BM cells in *Il2*^−/−^*Bim*^−/−^ mice showed normal pattern compared to the drastically reduced FSC^lo^SSC^lo^ BM cells in the *Il2*^−/−^ mice (Supplementary Figure 5C). Also, the spleen size (Figure 4M), and the distribution of lineage-positive cells in the *Il2*^−/−^*Bim*^−/−^ mice were comparable with that of control mice (Supplementary Figure 5D). Thus, our analysis shows that the lack of T_reg_ cell activity is detrimental for HSC maintenance and a T_reg_-Teff homeostasis is essential for normal hematopoiesis.

### Enhanced T_eff_ cell activity impairs HSC maintenance

**Figure 5:**
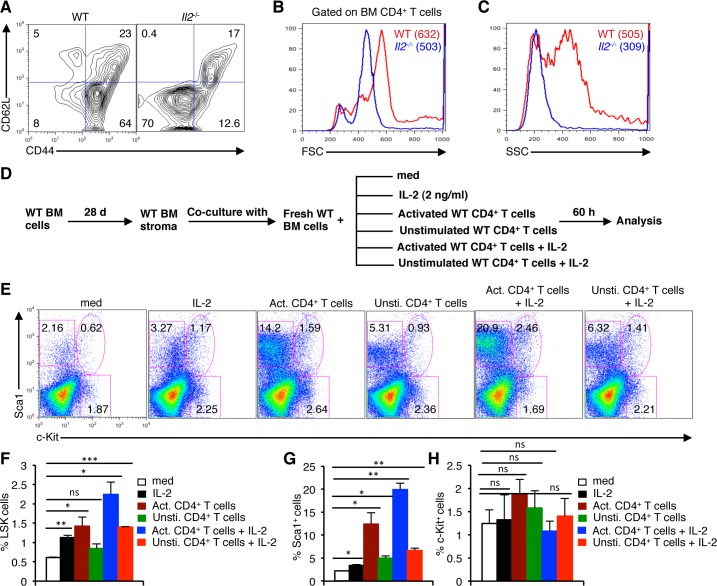
Activated T cells induce HSC defects in the BM **A**. Distribution of CD4^+^ T cells in the BM of *Il2*^−/−^ mice based on CD62L and CD44 expression compared to WT mice. **B**. Profiles of FSC distribution, and **C**. SSC distribution of the BM CD4^+^ T cells in WT and *Il2*^−/−^ mice. **D**. Experimental plan to analyze the involvement of activated CD4^+^ T cells in BM HSC maintenance. **E**. Flow cytometry profiles showing the influence of IL-2, of naïve or activated CD4^+^ T cells on the maintenance of HSCs in the BM cells-stromal cells co-culture assays. **F**. Quantification of LSK cells in the respective BM cells-stromal cells co-culture assays as indicated. **G**. Distribution of BM Lin^−^Sca1^+^, and **H**. Lin^−^c-Kit^+^ cells as evaluated from the BM cells-stromal cells co-culture assays in respective conditions as indicated. Numbers inside each dot plot represent percent respective population. Data are representative of 3 independent experiments, (*n* = 3 per group) and shown as mean ± s.d., in (F) **P = 0.0061, *P = 0.0185 or 0.0185 and ***P = 0.0002, (G) *P = 0.0135 or 0.0279 or 0.0143 and **P = 0.0029 or 0.0067, ns = not significant, one-way ANOVA.

As in steady state T_reg_ cells keep T_eff_ cell activity under control and avoid unnecessary T cell activation, we next investigated the phenotypes of T cells in the *Il2*^−/−^ mice. Similar to an earlier report [42], the majority of CD4^+^ T cells in the BM of *Il2*^−/−^ mice were of CD62L^−^CD44^−^ activated effector phenotype in contrast to the prevalence of CD62L^−^CD44^+^ effector memory cells in WT mice (Figure 5A). These CD4^+^CD62L^−^CD44^−^ T cells in the BM of *Il2*^−/−^ mice were smaller in size (Figure 5B) and much less granular (Figure 5C) compared to the WT cells.

To prove our assumption that activated CD4^+^ T cells can dysregulate HSC maintenance in the BM, we co-cultured WT BM cells with unstimulated, or PMA plus ionomycin stimulated WT CD4^+^ T cells (Supplementary Figure 6A), in presence or absence of IL-2 on WT BM stromal cell layer (Figure 5D). Interestingly, co-culture of BM cells with activated CD4^+^ T cells showed an increase in the proportion of LSK cells, which was further enhanced in the presence of IL-2 (Figure 5E, 5F) compared to the medium controls. Even IL-2 treatment alone, or in combination with unstimulated CD4^+^ T cells showed a significant increase in LSK cells (Figure 5E, 5F). Additionally, co-culture with activated CD4^+^ T cells also showed a strong increase in the Sca1^+^ cells similar to the phenotype in *Il2*^−/−^ mice, which was further increased in presence of IL-2 (Figure 5E, 5G). However, under each culture condition these anomalies in LSK and Sca1^+^ cells hardly affected the distribution of c-Kit^+^ cells (Figure 5E, 5H) suggesting that the increase in LSK cells is not due to a mere redistribution of Lin^−^c-Kit^+^ and Lin^−^Sca1^+^ cells.

### IL-10-independent defects in HSC maintenance in *Il2*^−/−^ mice

**Figure 6:**
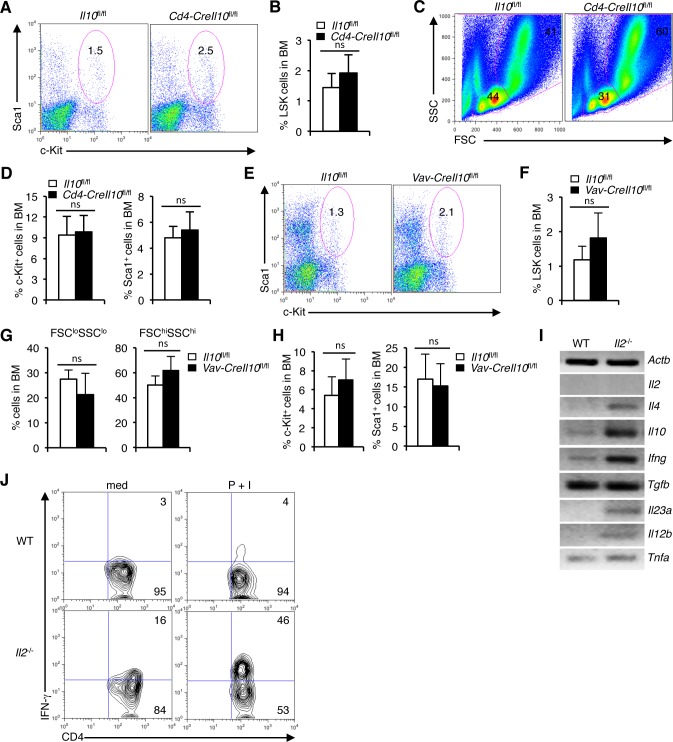
Dysregulated hematopoiesis in *Il2*^−/−^ mice is IL-10-independent **A**. Flow cytometry revealing the distribution of LSK cells in *Cd4*-Cre*Il10*^fl/fl^ mice compared to control mice. **B**. Proportion of LSK cells in the BM of *Cd4*-Cre*Il10*^fl/fl^ and *Il10*^fl/fl^ mice. **C**. Profiles of BM cells in *Cd4*-Cre*Il10*^fl/fl^ mice compared to control mice according to their FSC/SSC distribution pattern. **D**. Evaluation of c-Kit^+^ and Sca1^+^ cells distribution among lineage-negative BM cells in *Cd4*-Cre*Il10*^fl/fl^ and *Il10*^fl/fl^ mice. **E**. BM LSK cells distribution in *Vav*-Cre*Il10*^fl/fl^ mice compared to littermate control mice. **F**. Analysis of the proportion of BM LSK cells in *Vav*-Cre*Il10*^fl/fl^ and *Il10*^fl/fl^ mice. **G**. Evaluation of FSC^lo^SSC^lo^ and FSC^hi^SSC^hi^ cells in the BM of *Vav*-Cre*Il10*^fl/fl^ mice compared to *Il10*^fl/fl^ mice. **H**. Proportion of c-Kit^+^ and Sca1^+^ cells distribution among lineage-negative BM cells in *Vav*-Cre*Il10*^fl/fl^ and *Il10*^fl/fl^ mice. **I**. Semi-quantitative RT-PCR analysis of cytokine gene expression in *Il2*^−/−^ T cells compared to WT cells. **J**. Flow cytometry analysis of IFN-γ production by unstimulated or P+I stimulated CD4^+^ T cells in WT and *Il2*^−/−^ mice. Numbers inside each dot plot represent percent respective population and in histograms represent the MFI. Data are representative of 3 independent experiments, (*n* = 3 per group) and shown as mean ± s.d., ns = not significant, one-way ANOVA.

IL-10, produced by the T_reg_ cells is a key player in the T_reg_-mediated immune suppression [59–61]. Deficiency of IL-10 has been shown to induce anemia and other hematopoietic anomalies [62]. Therefore, we hypothesized that IL-10 deficiency in the *Il2*^−/−^ mice might contribute to the HSC defects as they have a strongly reduced T_reg_ population (Supplementary Figure 4A). To check if IL-10 influences HSC maintenance, we analysed *Cd4*^−^*CreIl10*^fl/fl^ mice in which IL-10 production was ablated not only in T_reg_ cells, but also in all T cells. Surprisingly, LSK cells distribution was unaffected in presence, or in T cell-specific loss of IL-10 activity (Figure 6A, 6B). A slightly decreased distribution of FSC^lo^SSC^lo^ BM cells (Figure 6C), and an unaffected Lin^−^c-Kit^+^ or Sca1^+^ cells was observed in the *Cd4-CreIl10*^fl/fl^ mice (Figure 6D). This was contrary to a recent report implicating IL-10 in maintaining HSC homeostasis [63]. To disprove the notion that IL-10 produced by other hematopoietic cells most likely compensated for the loss of IL-10 production by T cells in the *Cd4-CreIl10*^fl/fl^ mice, we ablated IL-10 production in all hematopoietic cells (*Vav-CreIl10*^fl/fl^ mice). Analysis of *Vav-CreIl10*^fl/fl^ mice showed a slight increase in BM LSK cells (Figure 6E, 6F), and an insignificant change in the distribution of BM FSC^lo^SSC^lo^ and FSC^hi^SSC^hi^ cells (Figure 6G). Also, the distribution of BM Lin^−^c-Kit^+^ or Sca1^+^ cells was unaffected in *Vav-CreIl10*^fl/fl^ mice compared to the WT controls (Figure 6H). The non-involvement of IL-10 in HSC maintenance was also clear as we observed a strong increase in IL-10 production in the *Il2*^−/−^ T cells (Figure 6I). Thus, despite having increased level of IL-10, HSC maintenance was drastically altered in the *Il2*^−/−^ mice.

To investigate what other factors produced by the activated T cells in the *Il2*^−/−^ mice could be involved in the hematopoietic abnormality, we analysed the expression pattern of several cytokines in these T cells. A strong increase in *Ifng* expression in addition to slightly increased expression of *Il4, Il12b, Il23a* and *Tnfa* was clearly evident in *Il2*^−/−^ mice compared to WT controls (Figure 6I). Compared to WT mice, upon stimulation, a huge increase in IFN-γ producing cells was observed in the *Il2*^−/−^ mice (Figure 6J). These observations suggest that an enhanced IFN-γ activity might be involved in the dysregulated hematopoiesis in *Il2*^−/−^ mice.

### Loss of IFN-γ activity rescues HSC defects in *Il2*^−/−^ mice

**Figure 7:**
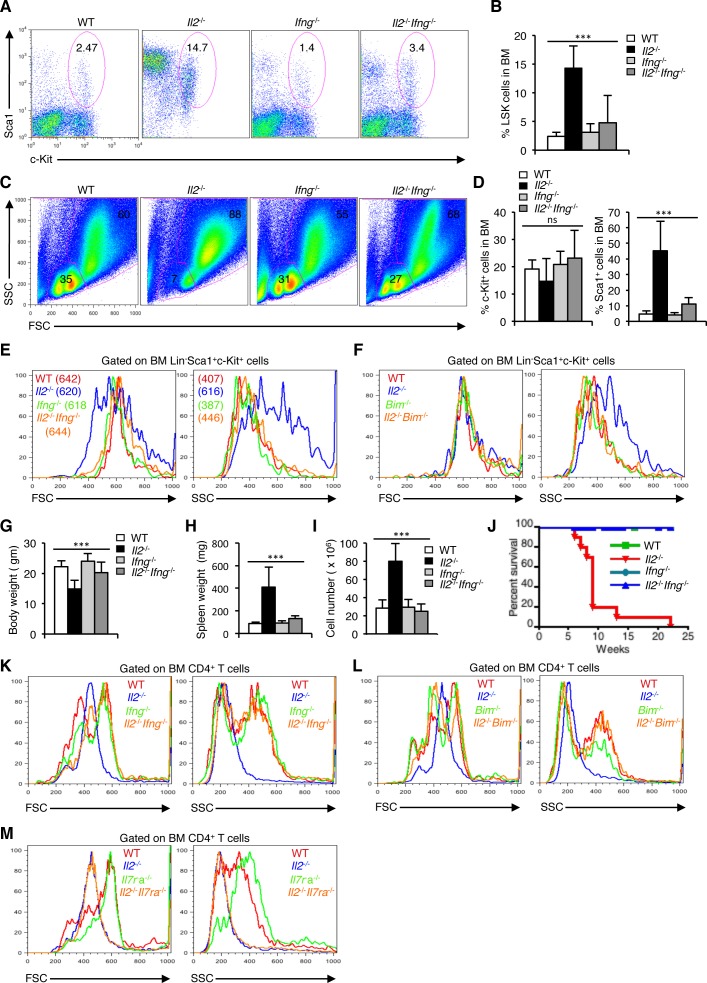
Enhanced IFN-γ activity leads to dysregulated hematopoiesis in *Il2*^−/−^ mice **A**. Distribution of LSK cells in BM from WT, *Il2*^−/−^, *Ifng*^−/−^ and *Il2*^−/−^*Ifng*^−/−^ mice. **B**. Quantification of BM LSK cells in *Il2*^−/−^*Ifng*^−/−^ mice compared to WT, *Il2*^−/−^and *Ifng*^−/−^ mice. **C**. Distribution of BM cells in indicated mice according to their FSC and SSC pattern. **D**. Evaluation of c-Kit^+^ and Sca1^+^ cells distribution among lineage-negative BM cells in *Il2*^−/−^*Ifng*^−/−^ mice compared to littermate control mice. **E**. FSC and SSC patterns of *Il2*^−/−^*Ifng*^−/−^ LSK cells compared to that of WT, *Il2*^−/−^ and *Ifng*^−/−^ cells. **F**. FSC and SSC patterns of *Il2*^−/−^*Bim*^−/−^ LSK cells compared to that of WT, *Il2*^−/−^ and *Bim*^−/−^ cells. **G**. Body weight, **H**. spleen weight, and **I**. splenocytes number in *Il2*^−/−^*Ifng*^−/−^ mice compared to WT, *Il2*^−/−^and *Ifng*^−/−^ mice. **J**. Survival curve for *Il2*^−/−^*Ifng*^−/−^ mice compared to WT, *Il2*^−/−^and *Ifng*^−/−^ mice. **K**. Profile of *Il2*^−/−^*Ifng*^−/−^ BM CD4^+^ T cells according to their FSC and SSC distribution pattern compared to that of littermate control mice. **L**. FSC and SSC distribution of *Il2*^−/−^*Bim*^−/−^ BM CD4^+^ T cells compared to that of littermate control mice. **M**. Profile of *Il2*^−/−^*Il7ra*^−/−^ BM CD4^+^ T cells according to their FSC and SSC distribution pattern compared to that of WT, *Il2*^−/−^and *Il7ra*^−/−^ mice. Numbers inside each dot plot represent percent respective population and in histograms represent the MFI. Data are representative of 3 independent experiments, (*n* = 3 per group) and shown as mean ± s.d., in B, D, G, H & I ***P < 0.0001, ns = not significant, one-way ANOVA.

To explore if IFN-γ is indeed involved in the dysregulated hematopoiesis, and whether by abolishing IFN-γ activity HSC maintenance could be restored in the *Il2*^−/−^ mice, we analysed the *Il2*^−/−^*Ifng*^−/−^ mice. In stark contrast to the *Il2*^−/−^ mice, LSK cells distribution in the *Il2*^−/−^*Ifng*^−/−^ mice was comparable with that of the littermate controls (Figure 7A, 7B). As a result, HSC maintenance was normal in the *Il2*^−/−^*Ifng*^−/−^ mice and the BM regained its usual distribution of FSC^lo^SSC^lo^ and FSC^hi^SSC^hi^ cells (Figure 7C). IFN-γ deficiency in the *Il2*^−/−^*Ifng*^−/−^ mice also reversed the high Sca1^+^ population observed in the *Il2*^−/−^ mice to WT levels without significantly affecting the c-Kit^+^ population (Figure 7D).

Abolition of IFN-γ activity not only restored the distribution of the LSK cells, but it also reversed the highly granular nature of *Il2*^−/−^ HSCs to that of WT levels in *Il2*^−/−^*Ifng*^−/−^ mice (Figure 7E). IFN-γ activity seems to be specifically influencing the granularity of HSCs, as the cell size was relatively unaffected in presence or absence of it (Figure 7E). In contrast, IFN-γ deficiency did neither influence cell size nor the granularity of Lin^−^Sca1^+^ or c-Kit^+^ cells (Supplementary Figure 6B, C). The beneficial effects of IFN-γ deficiency on HSCs in the *Il2*^−/−^ mice were comparable to that observed in case of *Il2*^−/−^*Bim*^−/−^ mice. Similar to the *Il2*^−/−^*Ifng*^−/−^ mice, LSK cells in *Il2*^−/−^*Bim*^−/−^ mice reverted back to the normal granularity when Treg activity was restored (Figure 7F). However, that was not the case in case of *Il2*^−/−^*Il7ra* mice, where the HSCs maintained higher granularity as in *Il2*^−/−^ mice (Supplementary Figure 6D), and the defects in hematopoiesis remained (Figure 3E, 3F and Supplementary Figure 3B, C). As a result of normal HSC activity, *Il2*^−/−^*Ifng*^−/−^ mice regained body weight (Figure 7G), and also these mice no more developed splenomegaly (Figure 7H, 7I). Significantly, IFN-γ-deficiency dramatically increased the life span of *Il2*^−/−^ mice, which otherwise died very early in their life (Figure 7J).

The hyperactive T cells in *Il2*^−/−^ mice BM were smaller and less granular compared to the littermate control mice, where majority of CD4^+^ T cells were larger and more granular (Figure 7K). Abolition of IFN-γ activity reversed the CD4^+^ T cell phenotype of *Il2*^−/−^ mice to that of WT mice (Figure 7K) suggesting this as an underlying mechanism, which facilitates HSC maintenance. In agreement with this, in *Il2*^−/−^*Bim*^−/−^ mice also the BM CD4^+^ T cells regained the phenotype as in case of littermate control mice (Figure 7L). However, in case of *Il2*^−/−^*Il7ra*^−/−^ mice, where HSC defects were still intact, the BM CD4^+^ T cells retained their smaller size and low granularity (Figure 7M). Thus, our study shows that abolition of IFN-γ activity in the absence of Treg cells (*Il2*^−/−^*Ifng*^−/−^), or restoring full Treg activity (*Il2*^−/−^*Bim*^−/−^) can reverse the abnormalities not only in HSCs but also in the CD4^+^ T cells in the BM, which were the prime cause of the hematopoietic abnormalities in *Il2*^−/−^ mice.

## DISCUSSION

We have previously reported a severely defective erythropoiesis in *Il2*^−/−^ mice due to the lack of KLF1 activity in erythroid precursor cells [42]. Also, earlier studies have reported severe autoimmunity, colitis and other pathological conditions in *Il2*^−/−^ mice [57, 64, 65]. However, defects at the level of HSC resulting in a highly dysregulated hematopoiesis threatening the survival of these animals have so far not been investigated. Maintenance of HSC quiescence and subsequent multiple lineage differentiation are governed by an array of signalling pathways and TFs. A role for IL-2 in HSC maintenance has not been elucidated though altered HSC distribution in these mice has been reported [66]. Here using multiple gene-manipulated mice we show that IL-2 signaling is an essential component of the complex regulatory mechanisms that need to be in place to maintain HSC integrity and steady state hematopoiesis.

Our findings of dysregulated Notch-Runx and other signalling molecules in HSCs lacking IL-2 signals is a critical attribute in explaining how the HSCs in *Il2*^−/−^ mice lose their quiescence and give rise to a myeloid-biased hematopoiesis. The HSCs in *Il2*^−/−^ mice are phenotypically distinct from that of WT mice, as they are highly granular (Figure 1D). This was a specific effect of IL-2 deficiency on HSCs only, as the Sca1^+^ or c-Kit^+^ cells were similar to that of WT cells, even though the Sca1^+^ population was strongly increased in *Il2*^−/−^ mice (Figure 1A). The observations regarding altered *Flk2* and *Slamf1* expression in *Il2*^−/−^ HSCs (Figure 1O), further vindicate an abnormal HSC maintenance in *Il2*^−/−^ mice. Besides, from our gene expression analysis of TFs and various signalling molecules involved in maintaining HSC quiescence (Figure 1P, 1Q), it is clear that the defects in *Il2*^−/−^ HSCs are specific defects arising out of IL-2 deficiency. The loss of FSC^lo^SSC^lo^ cells and the accumulation of FSC^hi^SSC^hi^ cells in the BM was a characteristic phenotype and could be used as a diagnostic trait for defective hematopoiesis in absence of IL-2 signals (Figure 1M, 1N). The fact that all these defects could be reversed with the treatment of IL-2 to the *Il2*^−/−^ mice (Figure 3A-3D and Supplementary Figure 3A) suggests that IL-2 signaling has an important contribution in maintaining HSCs in the BM. The hematopoietic phenotype observed in both our settings (Figure 2 and Supplementary Figure 2) at an early and late time points after adoptive transfer of BM cells, indicate that IL-2 signaling is critical in maintaining normal BM hematopoiesis.

Contrary to *Il2*^−/−^*Rag1*^−/−^ mice (Figure 3G, 3H and Supplementary Figure 3D, E) the persistence of the HSC defects in *Il2*^−/−^*Il7ra*^−/−^ mice (Figure 3E, 3F and Supplementary Figure 3B, C) underlines the important role T cells play in the maintenance of HSCs. The phenotype of T cells in the BM of *Il2*^−/−^ mice is very distinct compared to that in WT mice (Figure 5A), and we have previously reported that these activated T cells in the *Il2*^−/−^ mice cause severe defects in erythrocyte development [42]. Here also, we have unravelled a link between intact T_reg_ activity establishing control over T_eff_ cell activities, which is absolutely necessary for maintaining HSC integrity and normal hematopoiesis. Lack of optimal T_reg_ population in the *Il2*^−/−^ mice initiates the cascading effect of activated T_eff_ cells producing excess amount of IFN-γ, which in turn destabilizes the HSC physiology leading to defective hematopoiesis. Involvement of the T_reg_ cells in maintaining HSC integrity and in hematopoiesis was clearly evident in our inducible T_reg_ depletion model (Figure 4A-4G). Further, our analysis of the *Il2*^−/−^*Bim*^−/−^ mice, where restoration of T_reg_ activity prevented the loss of HSC quiescence and maintained hematopoiesis (Figure 4K, 4L and Supplementary Figure 5), suggests that the T_reg_ cells could actually fix the hematopoietic defects in *Il2*^−/−^ mice.

How do the T_reg_ cells exert such a profound influence on HSC integrity? T_reg_ cells are a prime source of IL-10, and a recent study has implicated IL-10 signaling to be critical for maintaining HSC quiescence [60, 61, 63]. However, in addition to the normal HSC phenotype and hematopoiesis observed in the *Cd4*-Cre*Il10*^fl/fl^ and *Vav*-Cre*Il10*^fl/fl^ mice (Figure 6A-6H), our finding of an increased IL-10 production in the *Il2*^−/−^ mice (Figure 6I) suggests that the HSC defects in these mice are IL-10-independent. On the other hand, the reversal in HSC defects in *Il2*^−/−^*Ifng*^−/−^ mice (Figure 7) demonstrated that increased IFN-γ activity was the main reason behind the dysregulated hematopoiesis in *Il2*^−/−^ mice. Our study unravelled the contrasting activities of IL-2 and IFN-γ, produced by the lymphocytes on HSC physiology and on hematopoiesis. IL-2 being the guarantor of T_reg_ survival and activity keeps the T cell activation in the BM under check resulting in minimal IFN-γ in the HSC niche. This in turn ensures optimal Notch-Runx signalling maintaining the HSC quiescence and normal hematopoiesis (Supplementary Figure 7). Any defects in IL-2 signaling will rapidly lead to T_reg_ cell death and breaking the delicate balance between T_reg_ and T_eff_ cell activity resulting in an excess IFN-γ having detrimental effect on hematopoiesis (Supplementary Figure 7). IFN-γ signalling has been reported to play a role in HSC maintenance and our study further supports this activity [67–69].

Our finding that an optimal number of T_reg_ cells are critical for HSC integrity will have wide impact in the clinical contexts of HSC reconstitution. Hematopoietic dysfunction is common following radio- or chemotherapy of patients suffering from various forms of cancer. Further, the observation regarding abolition of IFN-γ activity restoring hematopoiesis even in the absence of T_reg_ cells will foster the use of anti-IFN-γ in facilitating hematopoietic reconstitution in varied settings. In addition, while deciding on clinical remedies in the context of hematopoietic reconstitution the contrasting activities of both IL-2 and IFN-γ need to be carefully evaluated keeping in mind their important role in T cell function.

## MATERIALS AND METHODS

### Mice

C57BL/6 wild-type, *Il2*^−/−^, *Il2ra*^−/−^, *Il7ra*^−/−^, *Jak3*^−/−^, *Rag1*^−/−^, *Bim*^−/−^, DEREG, *Il2*^−/−^*Il7ra*^−/−^, *Il2*^−/−^*Rag1*^−/−^, *Il2*^−/−^*Bim*^−/−^, *Il10*^fl/fl^, *Cd4*-Cre*Il10*^fl/fl^, *Vav*-Cre*Il10*^fl/fl^, *Ifng*^−/−^ and *Il2*^−/−^*Ifng*^−/−^ mice of 6-8 weeks age unless mentioned otherwise were used in this study. All mice used in the study were on C57BL/6 background. Animals were housed either in the central animal facility (ZEMM) or in the animal facility of the Institute of Virology and Immunobiology, University of Würzburg, according to standard animal care protocols. All animal experiments were performed with extreme care, and were according to established guidelines (approved by the Regierung von Unterfranken, Wuerzburg, Permit Number 55.2-2531.01-53/10B).

### Flow cytometry

All antibodies for flow cytometry and cell isolation were purchased either from BD Pharmingen or eBioscience. Anti-Ly-6A/E (Sca1; D7), anti-c-Kit (2B8), Biotin mouse lineage panel (# 559971), anti-CD45R/B220 (RA3-6B2), anti-CD3ε (145-2C11), anti-CD4 (GK1.5), anti-CD8α (53-6.7), anti-CD11b (M1/70), anti-CD11c (N418), anti-CD25 (PC61), anti-CD41 (MWReg30), anti-CD44 (IM7), anti-CD45.1 (A20), anti-CD45.2 (104), anti-CD48 (HM48-1), anti-CD62L (MEL-14), anti-Ly-6G (Gr1; RB6-8C5), anti-F4/80 (BM8), anti-NK1.1 (PK136), anti-Ter119 (TER-119), anti-CD127 (A7R34), anti-CD135 (Flk2; A2F10), anti-CD150 (9D1), anti-Foxp3 (FJK-16s), anti-IFN-γ (XMG1.2), anti-IgM (R6-60.2) and anti-IgD (11-26c), antibodies either directly conjugated with fluorochromes or with biotin were used throughout this study. Biotinylated antibodies were revealed with secondary streptavidin-allophycocyanin or phycoerythrin-Cy5 (PE-Cy5) antibodies. HSC (LSK) population in BM was analyzed by gating either on lineage-negative cells not expressing IL-7Rα and high levels of both Sca1 and c-Kit molecules, or on LSK cells negative for CD48 and positive for high levels of CD150 expression. LT- and ST-HSCs were analyzed by further gating on LSK cells for Flk2 (CD135) expression. Flow cytometry and data analysis were performed following standard procedure using FACSCalibur and CellQuest or FlowJo software.

### Cell sorting

For cell sorting, femur and tibia from both hind limbs were collected and the bone marrow was flushed out by pumping BSS containing 0.1% BSA with the help of a syringe and needle. Single cell suspension was prepared and cells were washed once in BSS/BSA and then with PBS/0.1% BSA. Afterwards, total BM cells were incubated with biotinylated antibodies against lineage markers (anti-CD3, anti-B220, anti-CD11b/CD11c, anti-Gr1, and anti-Ter119) followed by incubation with anti-biotin microbeads. Lineage-positive and lineage-negative cells were isolated by magnetic separation. Lineage-negative fraction was further incubated with anti-IL-7Rα (CD127), anti-Sca1, anti-c-Kit and anti-Flk2 antibodies for sorting of LSK cells, or the LT- and ST-HSCs. Cells were sorted by using a FACSAria (BD Biosciences) flow cytometer.

### Intracellular staining

For intracellular IFN-γ staining, 5 × 10^6^ total BM cells from WT and *Il2*^−/−^ mice were either cultured in complete-RPMI (10% FCS) medium or stimulated with PMA plus Ionomycin (100 ng each) for 5 h in presence of GolgiPlug (1:1000 dilution) and Monensin (1:1500 dilution). For intracellular Foxp3 staining, freshly isolated 5 × 10^6^ splenocytes from WT and *Il2*^−/−^ mice, or BM, spleen and LN cells from PBS or DT treated DEREG mice were used. Cells were first surface stained for CD4, CD8 and CD25 followed by intracellular IFN-γ or Foxp3 staining according to eBioscience Foxp3 staining protocol. IFN-γ expression in the BM was analyzed by gating on CD4^+^ T cells. Similarly, Foxp3 expression in each case was analyzed by gating on CD4^+^CD25^+^ T cells (WT and *Il2*^−/−^ mice) or by evaluating GFP levels in DEREG mice. Flow cytometry was performed using a FACSCalibur (BD Biosciences) and data were analyzed using FlowJo software.

### Photographs

Photograph of spleens were taken using a Nikon Coolpix 4500 digital camera, and the photographs were processed using Adobe Photoshop software.

### Adoptive cell transfer

Single cell suspensions of BM cells were prepared and RBCs were lysed before transfer. For adoptive transfer, in the first set of experiments 4 × 10^6^ BM cells from CD45.2^+^ WT or *Il2*^−/−^ donors were transferred to lethally (9 Gy) irradiated CD45.1^+^ congenic WT recipients. In the reverse set of experiments BM cells from CD45.1^+^ WT donors were transferred to lethally irradiated CD45.2^+^ WT or *Il2*^−/−^ recipients by retro-orbital injection of the venous sinus. Post-cell transfer, recipient mice were maintained with antibiotic-supplemented drinking water and were treated with extra care. BM hematopoiesis was analyzed at 5 and 11 weeks (CD45.2^+^ WT or *Il2*^−/−^ donor cells), or at 4 and 13 weeks (CD45.1^+^ WT donor cells) after transfer by gating on donor-derived cells in each set of experiments. For the transfer of naïve CD4^+^ T_eff_ cells, 2.0 × 10^6^ CD4^+^CD25^−^CD62L^hi^ cells from LN and spleen were transferred intraperitoneally to *Rag1*^−/−^ mice, and, three weeks post-transfer, frequency of LSK cells in the BM was analyzed.

### *In vivo* injections

6-8 weeks old *Il2*^−/−^ mice were injected every alternate day with 1 μg recombinant murine IL-2 (rmIL-2, Peprotech) or an equal volume of PBS intraperitoneally for two weeks. Mice were analyzed to investigate the effects of rmIL-2 on BM hematopoiesis four days after the last injection. DEREG mice of 6 weeks of age were either injected with 1 μg (100 μl) diphtheria toxin (DT) or an equal volume of PBS intraperitoneally for four consecutive days. Subsequently, three days after last injection mice were analyzed for the effect of lack of T_reg_ cell activity on BM hematopoiesis.

### *In vitro* co-culture assay

Establishment of BM stromal cell layer: 20 × 10^6^ total BM cells from WT mice were plated into each well of a 24-well plate in 2 ml complete-RPMI (10% FCS) medium. Cells were incubated at 37°C and after 3 days all floating cells were removed and the adherent stromal cells were washed with complete-RPMI (10% FCS) medium. Cells were washed every 4 days and fresh medium was added to allow the stromal cells to grow up to confluency. At day 28, 5 × 10^6^ freshly isolated WT BM cells were added to the stromal cell layer and were co-cultured with 4 × 10^5^ unstimulated or 6 h PMA plus ionomycin stimulated WT CD4^+^ T cells in presence or absence of IL-2 (2 ng/ml). After 60 h, the cultures were analyzed to evaluate the influence of T cell activity on LSK cells in respective culture condition.

### Semiquantitative RT-PCR

Sorted LSK cells were used to synthesize cDNA using Miltenyi Biotec cDNA synthesis kit and protocol. Semiquantitative RT-PCR was performed to analyze the expression of indicated genes. Primer sequences are available in the supplementary information online.

### Statistics

Results are presented as mean ± s.d. Statistical significance was assessed using Student's *t*-test for comparison between two groups and ANOVA for the differences between groups.

## SUPPLEMENTARY MATERIALS FIGURES AND TABLES


