# Supplementary material for: Interleukin-2-regulatory T cell axis critically regulates maintenance of hematopoietic stem cells
Source: Oncotarget. 2017 Mar 18;8(18):29625–42. doi: 10.18632/oncotarget.16377 (PMC5444691; doi:10.18632/oncotarget.16377)
Supplement: Supplementary file 1 [file oncotarget-08-29625-s001.pdf]

Interleukin-2-regulatory T cell axis critically regulates maintenance of hematopoietic stem cells

Supplementary Material

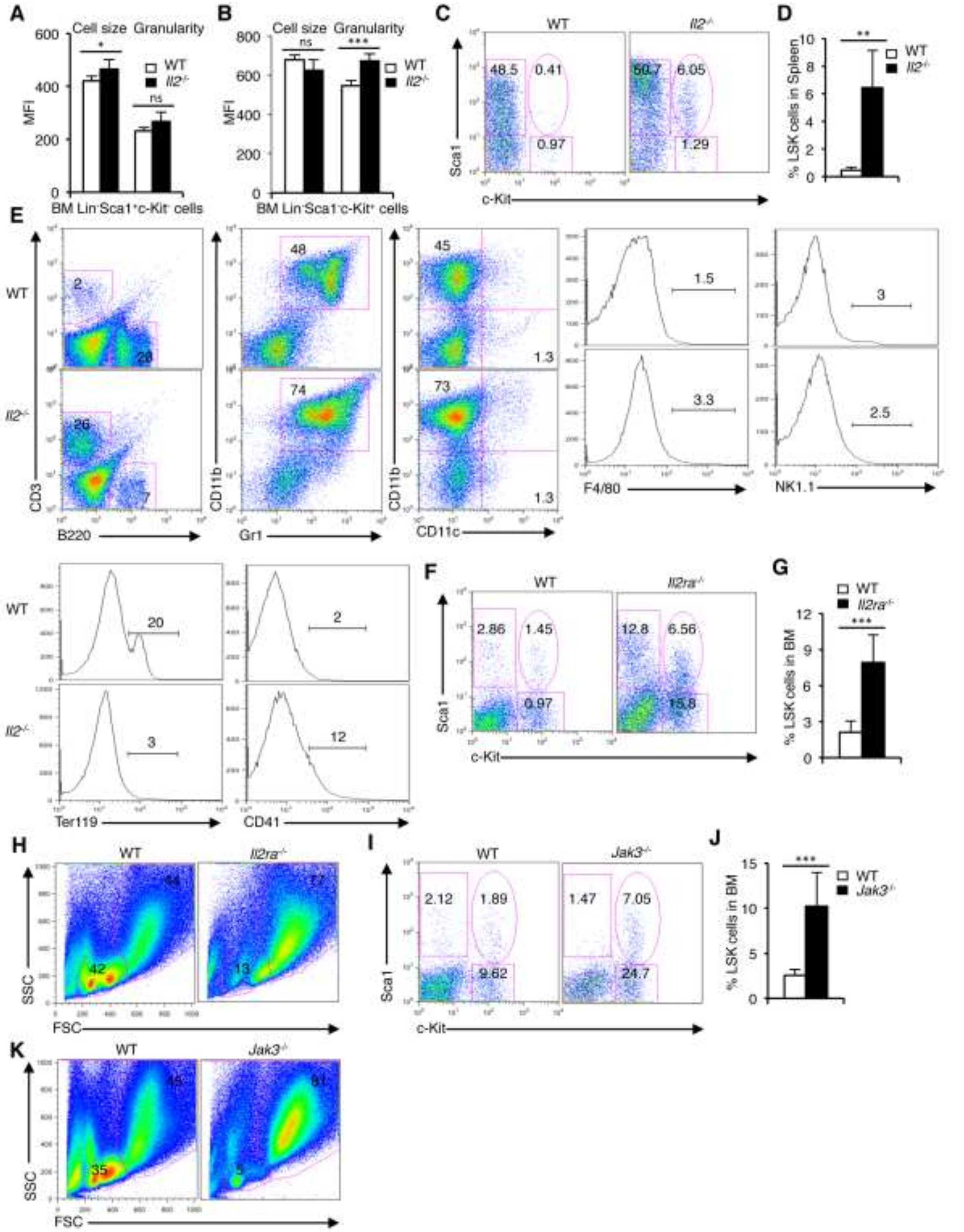

Supplementary Figure 1: Impaired HSC maintenance in IL-2 signaling-deficient mice. **A.** Evaluation of cell size and granularity of BM $Lin^-Sca1^+c\text{-}Kit^-$ cells from WT and *Il2*$^{-/-}$ mice. **B**. Cell size and granularity of *Il2*$^{-/-}$ BM $Lin^-Sca1^-c\text{-}Kit^+$ cells compared to WT cells. **C.** Flow cytometry profiles of LSK cells distribution among splenocytes in *Il2*$^{-/-}$ mice compared to WT mice. **D.** Quantification of percent LSK cells in the spleen of WT and *Il2*$^{-/-}$ mice. **E.** Distribution of lymphoid ($CD3^+$, $B220^+$ and $NK1.1^+$), myeloid ($Gr1^+$, $CD11b^+$, $CD11c^+$ and $F4/80^+$) and erythroid ($Ter119^+$ and $CD41^+$) cells in the BM of *Il2*$^{-/-}$ mice compared to WT mice. **F.** Distribution of LSK cells in the BM of *Il2ra*$^{-/-}$ mice compared to littermate WT controls. **G**. Quantification of percent LSK cells in the BM of WT and *Il2ra*$^{-/-}$ mice. **H.** FSC/SSC distribution pattern of BM cells from *Il2ra*$^{-/-}$ mice compared to WT control mice. **I.** LSK cells distribution in the BM of *Jak3*$^{-/-}$ mice compared to littermate WT controls. **J.** Quantification of percent LSK cells in the BM of WT and *Jak3*$^{-/-}$ mice. **K.** FSC/SSC distribution pattern of BM cells in WT and *Jak3*$^{-/-}$ mice. Numbers inside each FACS plot represent percent respective population. Data are shown as mean ± s.d., in (**A**) *P = 0.0439, (**B**) ***P = 0.0002, (**D**) **P < 0.0012, (**G**) ***P < 0.0001 and in (**J**) ***P = 0.0006, ns = not significant, unpaired *t*-test and are representative of 5 independent experiments, (n = 3 per group).

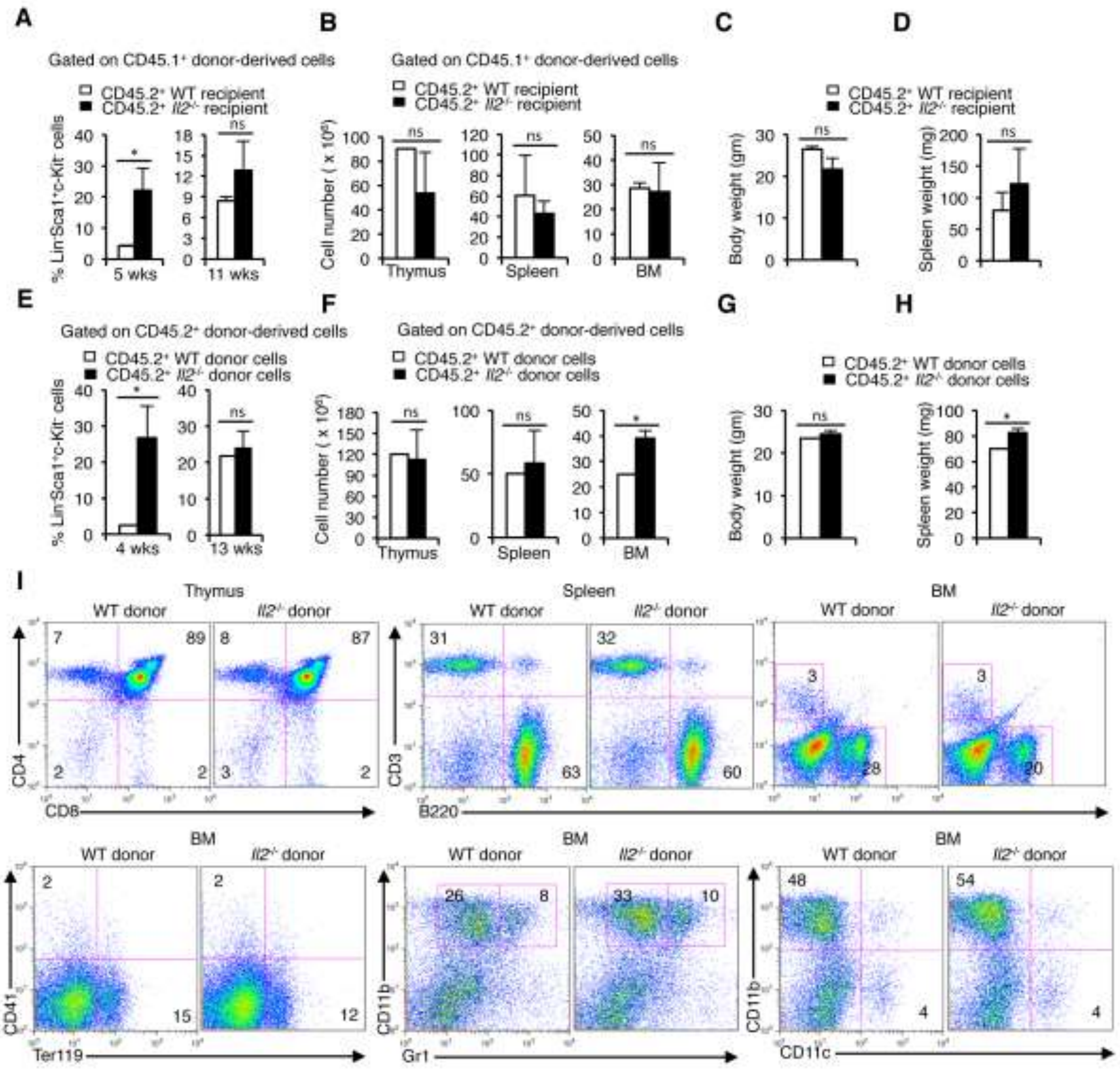

Supplementary Figure 2: Impaired reconstitution potential of *Il2*$^{-/-}$ HSCs. **A**. Evaluation of CD45.1^{+} donor-derived BM Lin^{-}Sca1^{+}c-Kit^{-} cells distribution in CD45.2^{+} WT or *Il2*$^{-/-}$ recipient mice 5 or 11 weeks after cell transfer. **B.** Cellularity in the thymus, spleen and BM of irradiated CD45.2^{+} WT or *Il2*$^{-/-}$ recipient mice 11 weeks after transfer of CD45.1^{+} WT BM cells. **C.** Evaluation of body weight of irradiated CD45.2^{+} WT or *Il2*$^{-/-}$ recipient mice 11 weeks after transfer of CD45.1^{+} WT BM cells. **D.** Spleen weight in CD45.2^{+} WT or *Il2*$^{-/-}$ recipient mice 11 weeks after transfer of CD45.1^{+} WT BM cells. **E.** Evaluation of CD45.2^{+} WT or *Il2*$^{-/-}$ donor-derived BM Lin^{-}Sca1^{+}c-Kit^{-} cells distribution in CD45.1^{+} WT recipient mice 4 or 13 weeks after cell transfer. **F.** Cellularity in the thymus, spleen and BM of

irradiated CD45.1$^+$ WT recipient mice 13 weeks after transfer of CD45.2$^+$ WT or *Il2*$^{-/-}$ BM cells. **G.** Evaluation of body weight of irradiated CD45.1$^+$ WT recipient mice 13 weeks after transfer of CD45.2$^+$ WT or *Il2*$^{-/-}$ BM cells. **H.** Spleen weight in CD45.1$^+$ WT recipient mice 13 weeks after transfer of CD45.2$^+$ WT or *Il2*$^{-/-}$ BM cells. **I.** Flow cytometry profiles of the distribution of various CD45.2$^+$ WT or *Il2*$^{-/-}$ donor-derived mature hematopoietic cell populations in the thymus, spleen and BM of CD45.1$^+$ WT recipient mice 13 weeks after cell transfer. Numbers inside each FACS plot represent percent respective population. Data are representative of two independent experiments, (n = 4 per group). Data are shown as mean ± s.d., in (**A**) *P = 0.0410, (**E**) *P = 0.0345, (f) *P = 0.0103, (**H**) *P = 0.0319, ns = not significant, unpaired t-test.

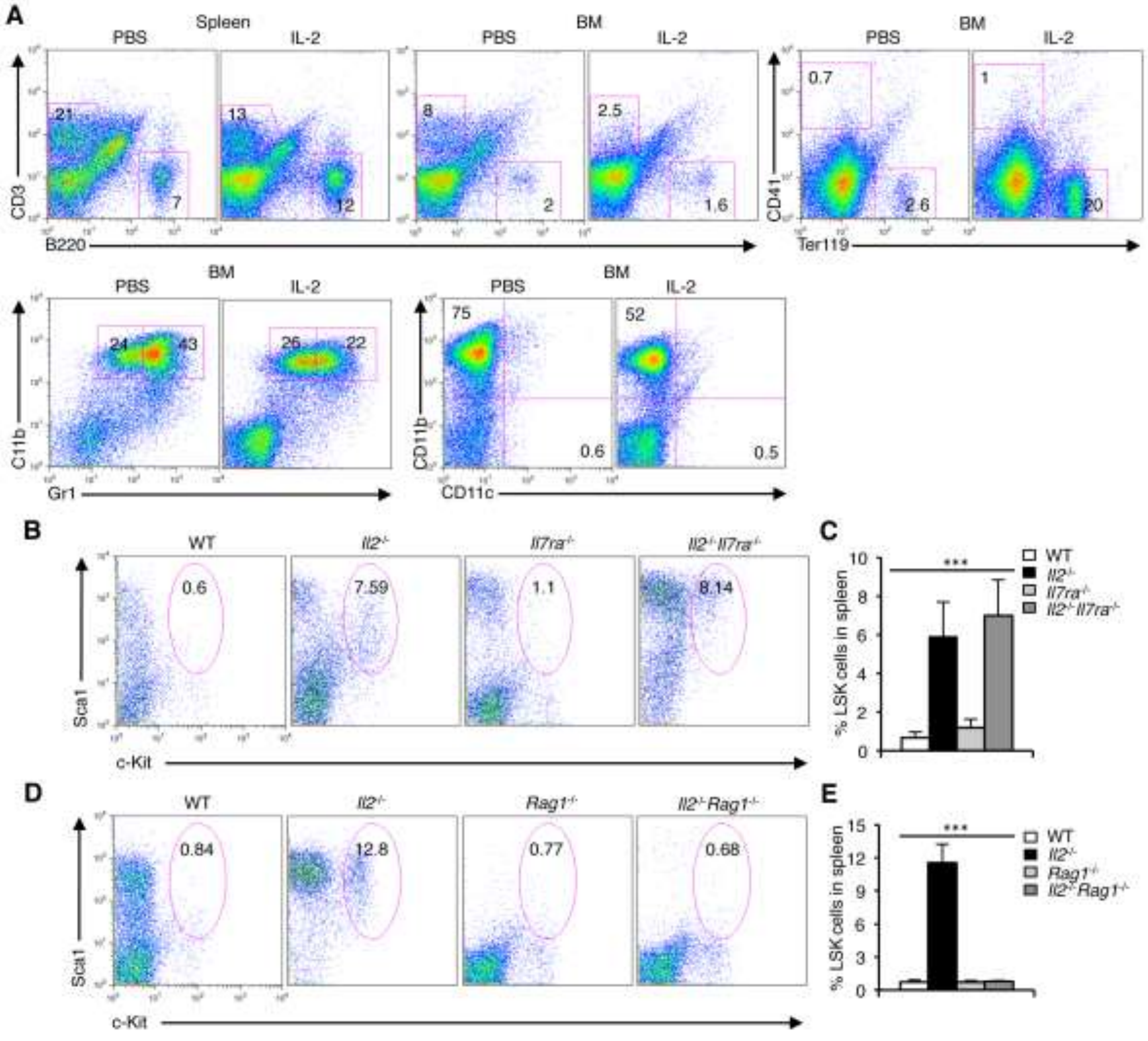

Supplementary Figure 3: T cell activity in the BM influences LSK cell maintenance. **A.** Distribution of lymphoid ($CD3^+$ and $B220^+$), erythroid ($Ter119^+$ and $CD41^+$) and myeloid ($Gr1^+$, $CD11b^+$ and $CD11c^+$) cells in the spleen and BM of PBS or IL-2 treated *Il2*$^{-/-}$ mice. **B.** Flow cytometry profiles showing the distribution of LSK cells in the spleen of *Il2*$^{-/-}$*Il7ra*$^{-/-}$ mice compared to WT, *Il2*$^{-/-}$ and *Il7ra*$^{-/-}$ mice. **C.** Quantification of percent LSK population in the spleen of indicated mice. **D.** Flow cytometry profiles showing the distribution of LSK cells in the spleen of *Il2*$^{-/-}$*Rag1*$^{-/-}$ mice compared to WT, *Il2*$^{-/-}$ and *Rag1*$^{-/-}$ mice. **E.** Quantification of percent LSK population in the spleen of indicated mice. Numbers inside each FACS plot represent percent respective population. Data are shown as mean ± s.d., ***P < 0.0001, one-way ANOVA, n = 4 per group, data represent one out of three independent experiments.

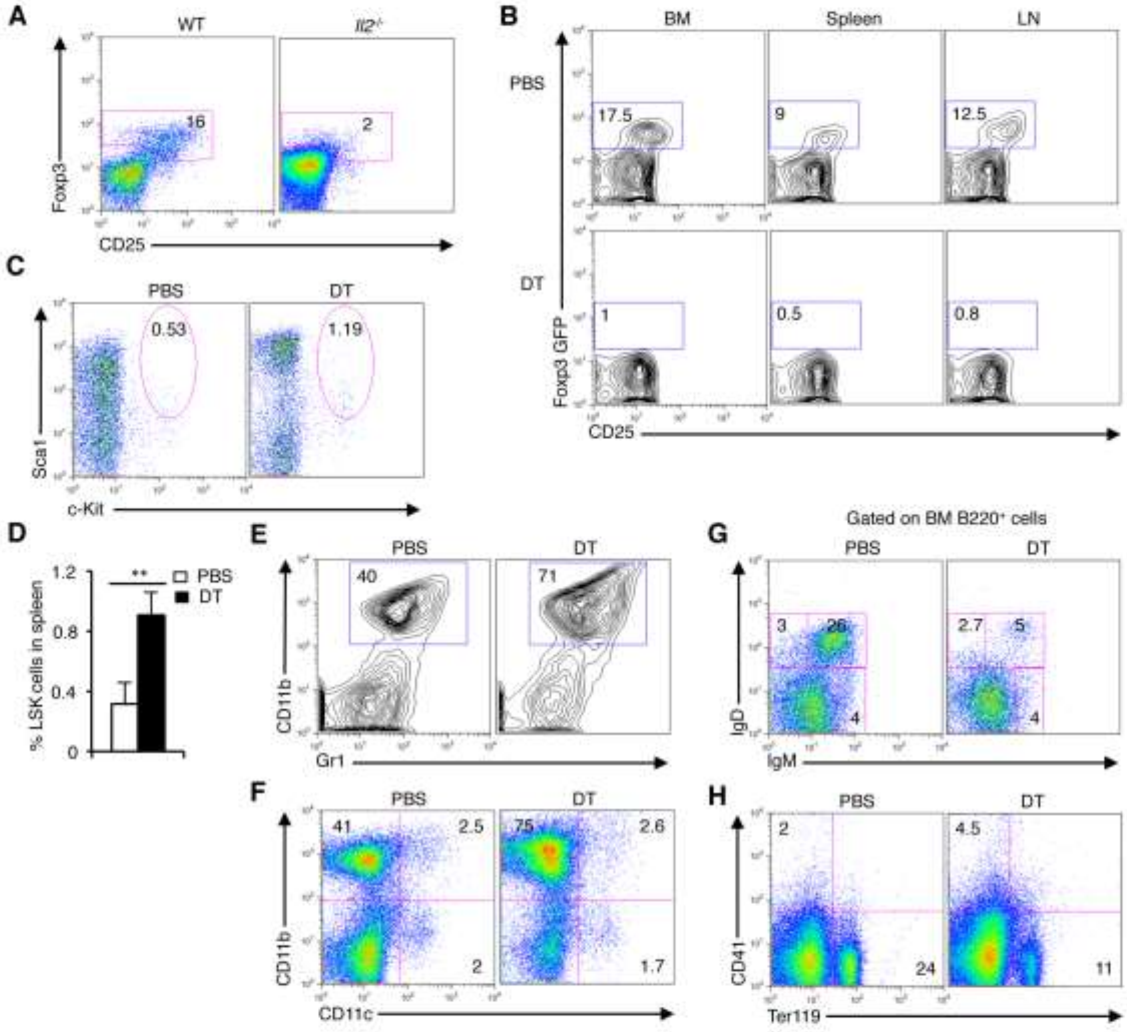

Supplementary Figure 4: T_{reg} cell activity is critical in maintaining BM hematopoiesis. **A.** Flow cytometry analysis of $CD4^{+}CD25^{+}Foxp3^{+}$ T_{reg} cell population in the spleen of WT or *Il2*$^{-/-}$ mice. **B.** Analysis of the distribution of $CD4^{+}CD25^{+}Foxp3^{+}$ T_{reg} cell population in the BM, spleen and LNs of PBS or DT treated DEREG mice. **C.** Distribution of LSK cells in the spleen of PBS or DT treated DEREG mice. **D.** Quantification of percent LSK population in the spleen of PBS or DT treated DEREG mice. **E-H.** Distribution of $CD11b^{+}$ and $Gr1^{+}$ cells (**E**), $CD11b^{+}$ and $CD11c^{+}$ cells (**F**), IgM^{+} and IgD^{+} cells (**G**), and $Ter119^{+}$ and $CD41^{+}$ cells (**H**) in the BM of DEREG mice treated with PBS or DT. Numbers inside each FACS plot represent percent respective population. Data are shown as mean ± s.d., $^{**}P = 0.0013$, unpaired *t*-test, $n = 4$ per group, data represent one out of three independent experiments.

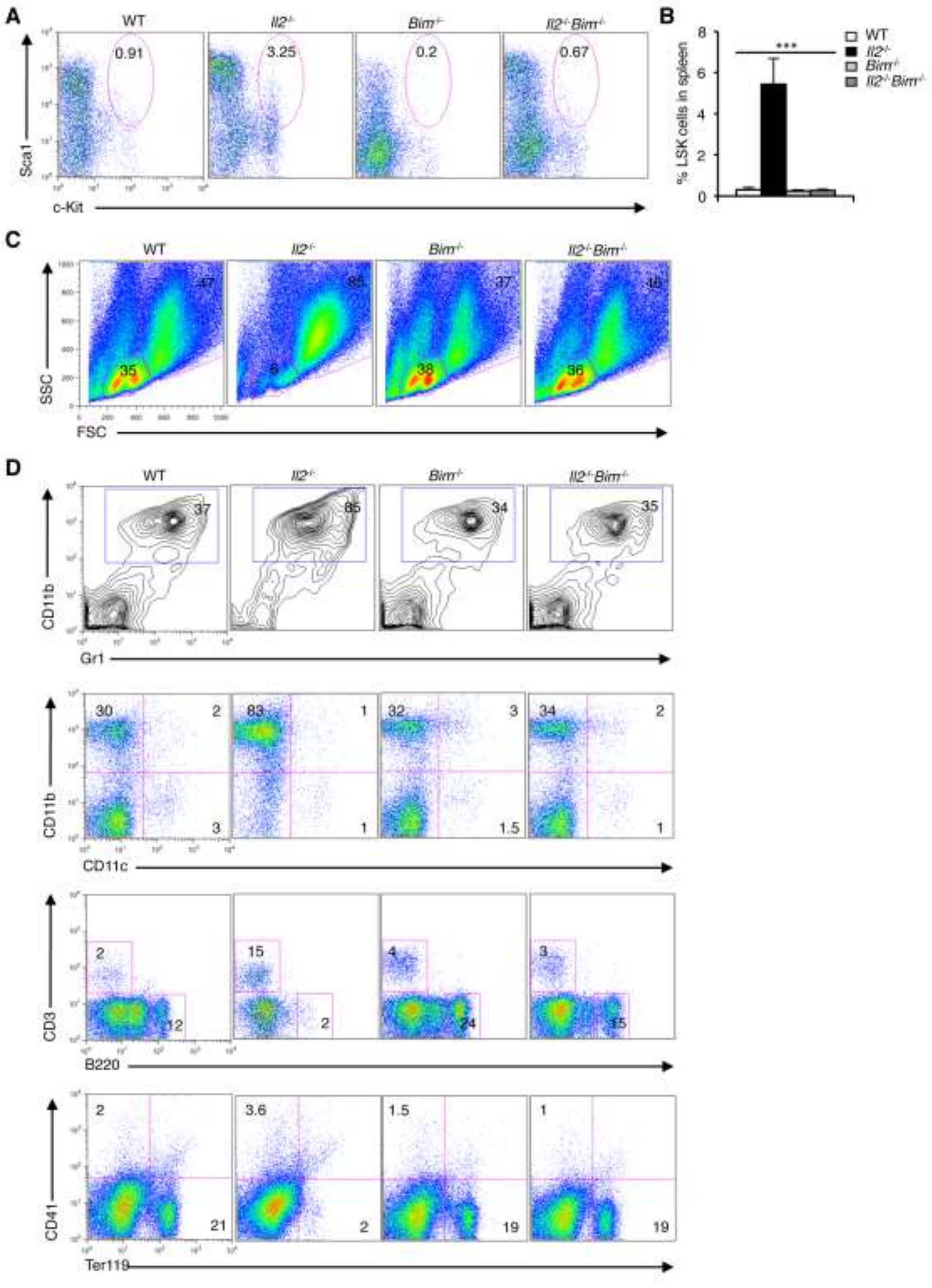

Supplementary Figure 5: Restoration of T_{reg} cells reverses hematopoietic defects in *Il2*$^{-/-}$ mice. A. Distribution of LSK cells in the spleen of *Il2*$^{-/-}$*Bim*$^{-/-}$ mice compared to WT, *Il2*$^{-/-}$ and *Bim*$^{-/-}$ mice as revealed by flow cytometry. **B.** Quantification of percent LSK population in the

spleen of indicated mice. **C.** Profiles of BM cells according to their FSC/SSC distribution pattern in *Il2*$^{-/-}$*Bim*$^{-/-}$ mice compared to WT, *Il2*$^{-/-}$ and *Bim*$^{-/-}$ mice. **D.** Distribution of myeloid (Gr1^{+}, CD11b^{+} and CD11c^{+}), lymphoid (CD3^{+} and B220^{+}), and erythroid (Ter119^{+} and CD41^{+}) cells in the BM of *Il2*$^{-/-}$*Bim*$^{-/-}$ mice compared to littermate control mice. Numbers inside each FACS plot represent percent respective population. Data are shown as mean ± s.d., ***P < 0.0001, one-way ANOVA, n = 4 per group, data represent one out of three independent experiments.

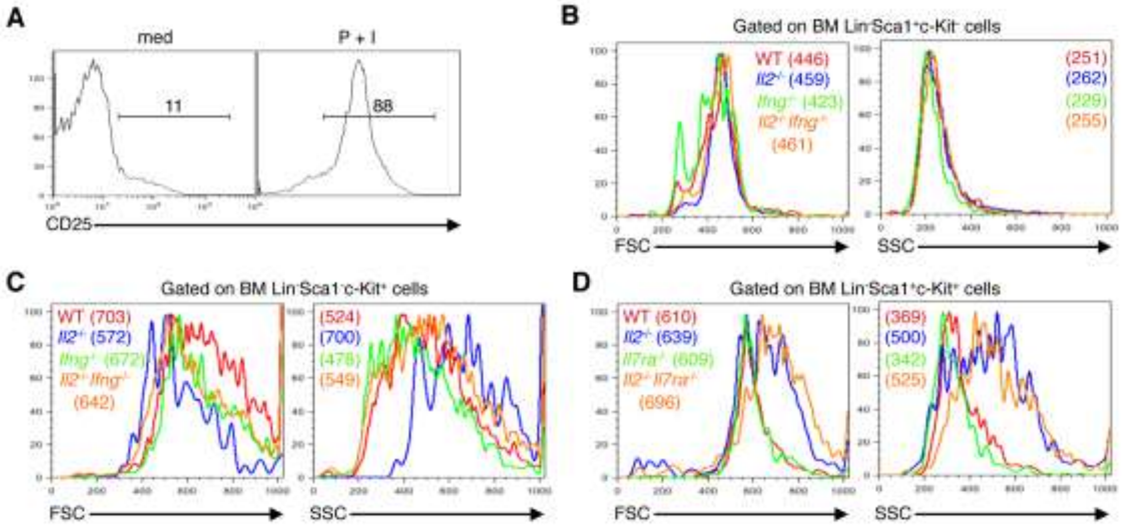

Supplementary Figure 6: **IFN-γ deficiency reverses HSC defects in *Il2*$^{-/-}$ mice**. **A.** Flow cytometry profiles of CD25 expression in WT CD4^{+} T cells either left unstimulated or stimulated for 6 h with P+I. **B.** FSC, and SSC profiles of BM Lin^{-}Sca1^{+}c-Kit^{-} cells from *Il2*$^{-/-}$*Ifng*$^{-/-}$ mice compared to WT, *Il2*$^{-/-}$and *Ifng*$^{-/-}$ mice. **C.** FSC, and SSC profiles of BM Lin^{-}Sca1^{-}c-Kit^{+} cells from *Il2*$^{-/-}$*Ifng*$^{-/-}$ mice compared to WT, *Il2*$^{-/-}$and *Ifng*$^{-/-}$ mice. **D.** Profile of BM LSK cells from *Il2*$^{-/-}$*Il7ra*$^{-/-}$ mice according to their FSC, and SSC distribution pattern compared to that from littermate WT, *Il2*$^{-/-}$and *Il7ra*$^{-/-}$ mice. Numbers inside the histograms in (**A**) represent percent respective population, and in the histograms in (**B-D**) represent MFI. Data represent one out of three independent experiments, n = 4 per group.

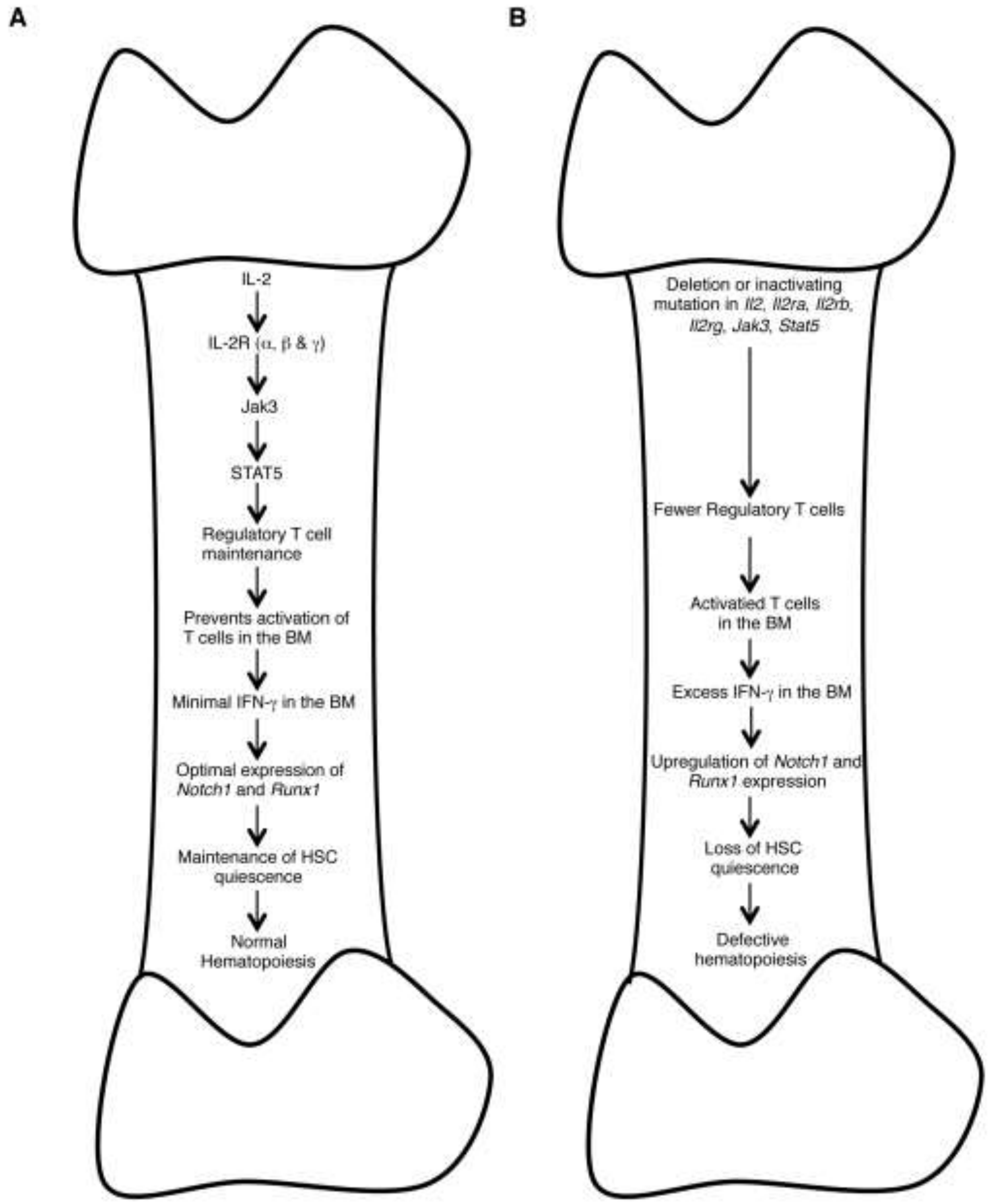

Supplementary Figure 7: **IL-2-dependent T_{reg} cell activity is essential for BM hematopoiesis**. **A.** Scheme showing the essentiality of IL-2 signaling on the maintenance of HSCs in the bone marrow. **B.** Schematic representation of the effects of deficiency in IL-2 or any of the components of IL-2 signaling pathway on HSC maintenance and hematopoiesis in the bone marrow.

Supplementary Table 1

LIST OF RT-PCR PRIMERS

Gene	Primer Sequence	Product Size
Actb	For: 5’-CCAGGTCATCACTATTGGCAAGGA-3’<br>Rev: 5’-GAGCAGTAATCTCCTTCTGCATCC-3’	223 bp
Bcl2	For: 5’-GGTGGTGGAGGAACTCTTCA-3’<br>Rev: 5’-CTCACTTGTGGCCCAGGTAT-3’	326 bp
Bcl2l1	For: 5’-TTCGGGATGGAGTAAACTGG-3’<br>Rev: 5’-TGTCTGGTCACTTCCGACTG-3’	318 bp
Bmi1	For: 5’-TGTCCTGTGTGGAGGGTACT-3’<br>Rev: 5’-CAACTTCTCCTCGGTCTTCA-3’	299 bp
Cdkn1b	For: 5’-GCTCTGCTCCATTTGACTGT-3’<br>Rev: 5’-GTCCCGGGTTAGTTCTTCAT-3’	293 bp
Cebpa	For: 5’-GGTGGACAAGAACAGCAACGAG-3’<br>Rev: 5’-TAGAGATCCAGCGACCCGAAAC-3’	325 bp
Csf1	For: 5’-AGTCTGTCTTCCACCTGCTG-3’<br>Rev: 5’-TGGTAGTGGTAGGCCACATT-3’	299 bp
Csf1r	For: 5’-ATGAGTCCCTCTTCACTCCG-3’<br>Rev: 5’-ACCTTCAGCACTGCATCTTC-3’	306 bp
Csf2ra	For: 5’-CCTGCTCTTCTCCACGCTAC-3’<br>Rev: 5’-ACTCCCCAGCAGTGACAAGT-3’	541 bp
Csf3r	For: 5’-AACTACACCCAGGCCTTCCT-3’<br>Rev: 5’-GAGCTCAAACTGGTCCTTGC-3’	606 bp
Cxcr4	For: 5’-GTGGATGGTGGTGTTTCAAT-3’<br>Rev: 5’-CTCGAAGTCACATCCTTGCT-3’	254 bp
Egr2	For: 5’-CTCCCATCTCTGCACCTAGA-3’<br>Rev: 5’-ATAAGGAGGAGGAGGTGGTG-3’	284 bp
Epor	For: 5’-GGACACCTACTTGGTATTGG-3’<br>Rev: 5’-GACGTTGTAGGCTGGAGTCC-3’	452 bp
Flk2	For: 5’- GACAAATCTCCCAATTGCAC -3’<br>Rev: 5’- GTGGCAGATCAACACAATGA -3’	291 bp
Gata1	For: 5’-ATTCCTGGGGGCTCACCTTATG-3’<br>Rev: 5’-TCCACAGTTCACACACTCTCTGGC-3’	366 bp
Gata2	For: 5’-AGCAAGGCTCGTTCCTGTTCAG-3’<br>Rev: 5’-CCATAAGGTGGTGGTTGTCGTC-3’	231 bp
Gfi1	For: 5’-GAGCTTCAAGAGGTCATCCA-3’<br>Rev: 5’-AGTCCATGCTGAGTCTCTCG-3’	306 bp
Hax1	For: 5’-GATGACGACGATGATGATGA-3’<br>Rev: 5’-CTGGGTTGGTGACTATCTGG-3’	330 bp
Hoxa9	For: 5’-TCACCAACCAAACACAACAG-3’<br>Rev: 5’-CACAATTAATCACGCCATCA-3’	299 bp
Hoxb4	For: 5’-TCACTCCCTTCCACCATAGA-3’<br>Rev: 5’-ATAACAACGGGAGAGGGTTC-3’	342 bp

Ifng	For: 5'-CAATGAACGCTACACACTGC-3'<br>Rev: 5'-CGCTTATGTTGTTGCTGATG-3'	279 bp
Il2	For: 5'-CCCTTGCTAATCACTCCTCA-3'<br>Rev: 5'-TTCCAATTGAAAGCTTTTGC-3'	392 bp
Il3ra	For: 5'-GCCCTGCATGGACAACACT-3'<br>Rev: 5'-GCACCGTAGCCACTGAAGTCA-3'	423 bp
Il4	For: 5'-GACGGCACAGAGCTATTGAT-3'<br>Rev: 5'-AAAATATGCGAAGCACCTTG-3'	253 bp
Il10	For: 5'-GGACCAGCTGGACAACATAC-3'<br>Rev: 5'-CACTCTTCACCTGCTCCACT-3'	263 bp
Il12b	For: 5'-TGTCTGCAGAGAAGGTCACA-3'<br>Rev: 5'-CAAAGGCTTCATCTGCAAGT-3'	218 bp
Il23a	For: 5'-GATCTGAGAAGCAGGGAACA-3'<br>Rev: 5'-CAGAACTGGCTGTTGTCCTT-3'	291 bp
Klf1	For: 5'-GATCGCCGGAGACGCAGGCT-3'<br>Rev: 5'-TCCCCAGTCCTTGTGCAGGA-3'	363 bp
Lef1	For: 5'-CCAGACTGTCTCCACAGCTT-3'<br>Rev: 5'-TGACAGGTCAGCACAGAAGA-3'	288 bp
Mpl	For: 5'-GAAATCTGCCTGCTGTGACT-3'<br>Rev: 5'-GAACCAGGAAGGAAGGTGAT-3'	254 bp
Meis1	For: 5'-AATGCCTATCGATTTGGTGA-3'<br>Rev: 5'-CCTTATCAGGGTCATCATCG-3'	266 bp
Myc	For: 5'-CATCCTGTCCATTCAAGC-3'<br>Rev: 5'-TAATTCCAGCGCATCAGT-3'	200 bp
Nfe2	For: 5'-GAGCCCTGGCCATGAAGATTCC-3'<br>Rev: 5'-CACCATCAGCAGCCTGTTGCAG-3'	391 bp
Notch1	For: 5'-TTGACGTCACTCTCCTGTGC-3'<br>Rev: 5'-ACACAGGTGCCATTGTTGAA-3'	408 bp
Notch2	For: 5'-ACCCTTGTATGCACGGAGTC-3'<br>Rev: 5'-CCAGGTTATTGCACGTTCCT-3'	373 bp
Pbx1	For: 5'-AAACTGCCACAGAATGAAGC-3'<br>Rev: 5'-CTTCTCCAGCTCTGTGTGGT-3'	298 bp
Pu.1	For: 5'-CGGATGACTTGGTTACTTACG-3'<br>Rev: 5'-GTAGGAAACCTGGTGACTGAG-3'	292 bp
Runx1	For: 5'-GAGGCAAACTCTGTCCTGAA-3'<br>Rev: 5'-TTAGGCCTCAAAGACACCTG-3'	312 bp
Scl	For: 5'-TTCTACAGGACGTGCTTTCC-3'<br>Rev: 5'-GAAACAGAAATGCCAGCCTA-3'	324 bp
Slamf1	For: 5'-TCCATGCCTCAGTTTCTCTC-3'<br>Rev: 5'-GGCAACTTTACAGCAGCATT-3'	264 bp
Tel/Etv6	For: 5'-TAAACACAGTTTGGCCCATT-3'<br>Rev: 5'-AACGCACAGCAGCTCTAACT-3'	273 bp
Tgfb	For: 5'-GCTTCAGCTCCACAGAGAAG-3'<br>Rev: 5'-CGTAGTAGACGATGGGCAGT-3'	255 bp
Tnf	For: 5'-TGGGTGTTCATCCATTCTCT-3'<br>Rev: 5'-TTTGAGTCCTTGATGGTGGT-3'	232 bp
Trp53	For: 5'-CAGGGCTGAGACACAATC-3'<br>Rev: 5'-TGGGGTAGGGTGAGATTT-3'	201 bp
